# Algae Protein Creates Sustainable Alternatives for Various Food Matrices: From Function to Nutrition

**DOI:** 10.1111/1541-4337.70264

**Published:** 2025-08-22

**Authors:** Shaozong Wu, Paul Menut, Song Miao, Christelle Turchiuli

**Affiliations:** ^1^ Université Paris‐Saclay, INRAE, AgroParisTech, UMR SayFood Palaiseau France; ^2^ Guangdong Provincial Key Laboratory of Food Quality and Safety, National‐Local Joint Engineering Research Center for Processing and Safety Control of Livestock and Poultry Products, College of Food Science South China Agricultural University Guangzhou P. R. China; ^3^ Teagasc Food Research Centre, Moorepark Cork Ireland

**Keywords:** algae, algae protein food, alternative protein, protein functionalities, protein processing, protein profile

## Abstract

Protein deficiency and environmental deterioration are pressing and complex issues in traditional agriculture system. Algae, which can grow without the need of land and with minimal water, offer a rich source of protein. Recently, large‐scale algae cultivation and advanced extraction techniques have been developed, positioning algae protein as a promising alternative to traditional animal proteins in various food categories. This review explores the global development of algae protein in the food industry, emphasizing its potential in association with animal protein or as a substitute for animal protein in foods. It highlights the importance of algae protein extraction and quality in food structuring and nutrition. Algae protein can be tailored to create a wide range of food products, though its properties are not fully understood and depend on cultivation conditions and extraction methods. Currently, the utilization of algae protein can be achieved through the use of entire biomass or of protein concentrates, which may contain a variety of proteins and non‐protein components. Despite the challenges associated with non‐purified algae protein, the field is advancing toward efficiently extracting protein from the algae matrix and incorporating it into new food matrices. This progress makes the application of algae protein in “blue foods” increasingly promising. However, like plant proteins, algae protein faces the dual challenges of sustainability and functionality.

## 1 Introduction

The relentless pursuit of new food sources and high‐yield agricultural production has been fundamental to the evolution of human civilization. Despite global hunger levels remaining stable in 2021 and 2022, the COVID‐19 pandemic exacerbated food insecurity, affecting 9.2% of the population in 2022 (UNICEF 2023). This highlights the fragility of the current global food supply chain and underlines the importance of alternative foods to supplement the food source. Protein, an essential nutrient for human health, is crucial for bodily functions and development. With the anticipated population growth, the global demand for protein is expected to surge, necessitating the expansion of food protein sources as well. Algae present a promising solution, being rich in protein and other micronutrients such as pigments, vitamins, and minerals. Compared to plant‐based proteins, algae protein offers a higher yield upon extraction (Benelhadj et al. 2016; Teuling et al. 2019). Meanwhile, algal peptides also possess various functional benefits (Custódio et al. 2012), as well as a balanced profile of essential amino acids, making it a suitable alternative protein source in food. The growing awareness of health benefits has significantly boosted the European algae market, which grew by approximately 43% from 2016 to 2023 and is projected to reach 1240 million euros, compared to a global market value of 4810 million euros in 2023 (Mendes et al. 2022).

Regarding the sustainability of algae as a food source, algae production is environmentally friendly due to its lower land and water requirements compared to animal protein production or plant protein cultivation. As a source of essential nutrients, algae can be cultivated at a lower cost compared to traditional farming. Additionally, algae demonstrate greater adaptability in farming compared to crops and livestock (Fu et al. 2021). Over 70% of the Earth's surface is covered by water bodies such as oceans, seas, rivers, and lakes (Water Science School 2019). Algae could survive well in these vast water areas of Earth. Remarkably, algae can also be cultivated in deserts, utilizing less valuable land for food production. However, challenges such as exposure to excessive UV light and high salt concentrations due to intense evaporation must be addressed (Sikkema 2021). Furthermore, algae can be integrated into urban agriculture without occupying arable land, which could contribute to carbon neutrality by converting atmospheric CO_2_ into carbohydrates, lipids, and other valuable bioproducts through photosynthesis (Sadvakasova et al. 2023). It is expected that the increased demand for algae in food production could help mitigate environmental greenhouse gas issues. Consequently, algae represent a sustainable protein source for humanity.

**FIGURE 1:**
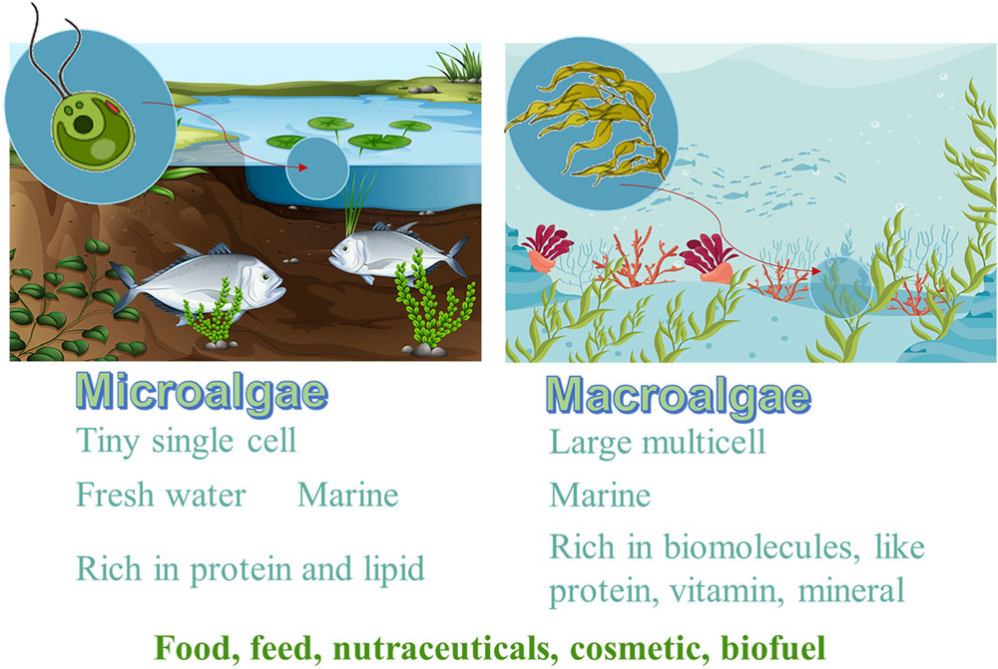
Comparison of the characteristics of microalgae and macroalgae (European Parliament 2023).

Microalgae are eukaryotic photosynthetic microorganisms, primarily unicellular plants, encompassing various types such as dinoflagellates, green algae (*Chlorophyta* or *Chlorophyceae*), golden algae (*Chrysophyceae*), and diatoms (Bacillariophyceae), each offering distinct benefits for humans. In contrast, macroalgae are multicellular plants, typically yielding higher outputs than terrestrial crops. Common macroalgae, such as seaweed, are rich in protein, minerals, and vitamins (Boukid et al. 2021; Packer et al. 2016) (Figure 1). Barba (2017) identified four major challenges for utilizing microalgae and seaweeds in food applications: defining molecules of interest, developing extraction techniques and processing methods, identifying the relationship between the algae matrix and yield, and optimizing algae extraction processes.

**FIGURE 2:**
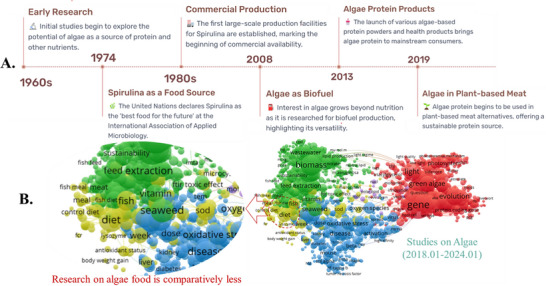
Algal protein development in the food sector analyzed by MyLens.AI in a topic “The Rise of Algae Protein in Human Nutrition” (A). Published studies in all research fields dealing with “algae protein” between January 2018 and January 2024 in the Web of Sciences (VOS VIEWER software) (red: biology; blue: healthiness; green: biomass production; yellow: diet) (B).

In recent years, the industrial use of algae has expanded significantly from animal feed to human food. Figure 2A illustrates the rapid development of algae protein in the food sector. From the 1960s to 2019, the algae industry evolved from initial discovery and scaling‐up phases to becoming a recognized source for health products since 2013. Subsequently, algae experienced a substantial leap in development as a potential ingredient in plant‐based food, such as in meat analogs. Although a number of research projects were conducted on algae between 2018 and 2024, it should be noted that those focusing on food applications are comparatively fewer, which suggests an important place for the study of algae for food purposes (Figure 2B). To foster the growth of the algae industry, global policies regarding algae food products have become more prevalent. Notably, algae species without a prior consumption record before 1997 require authorization, with food safety considerations in mind.

1. In EU, in 2022, 22 algae species were approved as novel food resources, but strict regulation of food safety issues, particularly contaminants and pathogens, remains essential (Su et al. 2023). In line with the Paris Agreement, the EU is leveraging the bioeconomy to reduce its carbon footprint, and European policies strongly support a sustainable food system to protect the environment and increase yields through alternative food sources. Thus, projects such as “Food and Natural Resources” have been funded under the Horizon Europe program (European Commission 2018). In 2020, European Commission has set a long‐term goal of achieving carbon neutrality by 2050 (Araújo et al. 2021).

2. As for Asia, Asians have a long‐standing tradition of algae consumption and are witnessing rapid growth in alternative food development from both marine sources and fermentation processes. Looking ahead, algae are expected to be consumed not only as a traditional dish but also as a novel protein source (Mendes et al. 2022). A significant policy introduced by the central government of China in 2023 emphasized the importance of diversifying the food supply system by developing microorganisms, edible fungi, and algae‐based foods alongside traditional plant and animal sources (Grahame 2023).

3. In the United States, New York Mayor Eric Adams has committed to reducing the city's food‐based emissions by 30% by 2030. The government is closely monitoring the safety of algae as a food source, where some are deemed Generally Recognized as Safe (GRAS) and some others are looking to be declared GRAS (Su et al. 2023).

Algae can function as an efficient bio‐factory for producing protein. Following cultivation and harvesting, algae undergo extraction processes to utilize their protein content effectively. This process involves complex steps of cell disruption and protein enrichment by mechanical, chemical, or biological methods. Isolated algae protein holds significant potential for expanding its application in food. Innovative extraction methods, such as ultrasound‐assisted extraction, pulsed electric fields, and microwave‐assisted extraction, enhance protein extraction quality compared to traditional methods. These new techniques improve the yield and quality of bioactive peptides, which have applications in human nutrition, animal feed, and aquaculture (Bleakley and Hayes 2017). The quality and quantity of algae protein significantly impact the final product, which is also determined by the target consumers. Several factors potentially influence the quality of algae protein: 1. anti‐nutritional factors like lectins; 2. cultivation conditions; 3. proximate biochemical composition; 4. type of algae species. Additionally, considerations such as protein content and amino acid profile, protein and essential amino acid digestibility, and biological performance evaluation of protein utilization are crucial (Bhatnagar et al. 2023).

To apply algae protein into food, it is necessary to clarify the functionalities of algae protein after extraction. The functionalities of algae protein encompass a wide range of properties, including solubility, emulsification, gelation, and foaming. These functionalities form the foundation for developing algae‐based food products and depend on the extraction processes applied to the algae protein. Despite significant challenges in optimizing these functionalities, advancing algae applications in food requires an interdisciplinary approach that integrates science, technology, engineering, and product processing. Although health concerns related to algae protein are important, the potential benefits of incorporating algae into various food matrices should not be overlooked. The limited current application of algae in food means that many of these benefits have not been fully explored or clarified (Ahmad and Ashraf 2023).

The objective of this review article is to provide an updated overview of the use of algae proteins for applications in the food industry, while highlighting the relationship between protein functional properties and nutrition. It will therefore:

1. Compare algae protein to other sources regarding the composition and amino acid profile;
2. Highlight the effects of environment and processing conditions on the functionality of protein;
3. Show the barriers for algae protein to partially or totally replace animal protein in food.

After highlighting the potential of algae proteins in food, we will focus on extraction methods, both from a qualitative and quantitative point of view, for the production of algae proteins with ideal functional and nutritional properties for use in food.

## 2 Literature Selection Criteria

The literature relating to this review article were collected comprehensively, especially the latest research about algae in food. The keywords searching the literatures were as follow: “algae,” “algae protein,” and “alternative protein.” An amount of 135 literatures were selected from the searching, where are at least 70 studies about algae protein, 16 studies about plant protein, 26 studies about animal protein, and the rest studies about the background of alternative protein. The important results of the literatures were discussed to enlighten the potentials of algae protein in food from function to nutrition. The reliability and quality of literatures and citation were ensured during the review article writing.

## 3 Protein and Amino Acid Profile of Algae

Algae protein is an ideal food source that meets both the quantity and quality requirements for human nutrition. It provides a rich supply of protein, along with a comprehensive amino acid profile that includes all essential amino acids.

### 3.1 Protein

The protein classes and types in algae are diverse and complex, similar to those found in leguminous seeds (Teuling et al. 2017). SDS‐PAGE analysis reveals that the protein composition of *Tetraselmis* sp. includes molecular weights of 50, 40, 25, and 15 kDa, with a distinct band observed at 50 kDa, whereas the bands smaller than 50 kDa could be the enzymes (e.g., RuBisCo) of polypeptide structure (Schwenzfeier et al. 2011). The soluble protein isolates of *Nannochloropsis gaditana* and *Tetraselmis impellucida* are abundant in RuBisCo, comprising approximately 20%–40% of the total protein. In contrast, the protein isolate from *Arthrospira maxima* is predominantly composed of C‐phycocyanin. Additionally, the protein isolates of *N. gaditana* and *T. impellucida* contain a higher proportion of multimeric proteins compared to *A. maxima*. Notably, high levels of monomeric proteins are found in these soluble protein isolates (Teuling et al. 2019). Generally, the protein content remains stable within the same algae strains. An important point to take into account for protein quantification is that compared to animal proteins, algae proteins contain a portion of non‐protein nitrogen. Recent studies indicate that non‐protein nitrogen contributes an average of 5.3% to the protein calculation (Sägesser et al. 2023).

### 3.2 Amino Acids

Amino acids, particularly essential amino acids, are fundamental for human protein synthesis and food formulation (Le Roux 2019). According to the FAO dietary requirements for adults (WHO 2007), alternative protein sources must provide a balanced amino acid profile. Algae proteins offer a well‐balanced profile of essential amino acids compared to certain plant, animal, and insect proteins (Table 1). For instance, the essential amino acid scores for *Chlorella* sp. and *Spirulina Bio* are 107.5 and 102.6, respectively (Mišurcová et al. 2014). For comparison, the amino acid scores of casein, minced beef, and soy protein are 99, 93, and 69, respectively (Friedman 1996). This indicates that their amino acid profiles are comparable to those of some animal proteins, such as milk protein (Fu et al. 2021). Regarding the distribution of amino acids within the algae matrix, non‐essential amino acids are predominantly located in the internal regions of the algae (Safi, Charton, et al. 2014). The overall amino acid content of brown seaweed *Himanthalia elongata* (Linnaeus) S. F. Gray is reported to be 54.02 ± 0.46 g/kg dry matter, with particularly high levels of lysine and methionine (Garcia‐Vaquero et al. 2017). Ideal amino acid profiles are characterized by high content and consistency. Two strains of *Galdieria sulphuraria* exhibit comparable amino acid compositions, demonstrating significant intraspecies biological conservation across different strains (Canelli et al. 2023). This high level of conservation is evident regardless of the trophic mode. Furthermore, the amino acid profiles of *G. sulphuraria* strains outperform those of some food‐grade primary algae (e.g., *Arthrospira* and *Chlorella*) and plant proteins (e.g., soybean) (Abiusi et al. 2022; Canelli et al. 2023). Thus, the quality of algae protein is closely related to the species and processing strategies employed (Table 2).

**TABLE 1:** Essential amino acids composition of some microalgae, macroalgae, animal, and insect food (DW%, on dry weight basis; %, on wet weight basis).

	**Categories**	Threonine	Valine	Methionine	Isoleucine	Leucine	Phenylalanine	Lysine	Histidine	Tryptophan	References
**Animal**	Bovine milk. %	0.24	0.23	0.14	0.19	0.42	0.23	0.38	0.24	∖	Landi et al. (2021)
	Angus Beef, %	0.92	0.82	0.50	0.79	1.34	0.79	1.78	0.91	0.41	Alekseeva and Kolchina (2019)
	Egg, %	0.613	0.7818	0.3943	0.6983	1.100	0.6798	0.923	0.2968	0.147	Attia et al. (2020)
	Chicken breast, %	1.05	1.02	1.06	0.83	1.51	0.79	2.12	0.74	0.44	Dalle Zotte et al. (2020)
**Microalgae**	*Spirulina* (*Arthrospira platensis*), DW%	3.961	2.789	0.807	2.534	3.969	1.902	2.262	2.739	∖	Raczyk et al. (2022)
	*Scenedesmus* sp., DW%	1.208	1.499	0.519	1.04	2.124	1.403	1.717	0.524	∖	Noreen et al. (2021)
	*Chlorella vulgaris*, DW%	1.268	1.571	0.549	1.011	2.29	1.45	1.698	0.561	∖	
	*Chlorella* sp., DW%	5.1	6.8	2.8	4.8	9.9	6.0	6.6	2.3	1.0	Pereira et al. (2019)
	*Tetraselmis* sp. CTP4, DW%	1.27	1.55	0.61	1.12	2.28	1.44	1.70	0.04	0.37	
	*Tetraselmis chui*, DW%	4.1	4.9	2.5	3.5	7.5	4.8	5.7	1.6	2.4	
	*Arthrospira* sp., DW%	5.1	6.0	2.0	5.5	8.5	4.8	5.2	1.9	1.6	
**Macroalgae**	*Saccharina japonica* (Kelp), DW%	∖	6.5	0.6	1.9	2.4	0.9	1.3	0.4	8	Nie et al. (2023)
	*Himanthalia elongata* (Sea Spaghetti), DW%	0.23	0.25	0.14	0.20	0.35	0.30	0.31	0.09	∖	Mohammed et al. (2021)
	*Alaria esculenta* (Irish wakame), DW%	0.50	0.53	0.13	0.39	0.74	0.51	0.57	0.13	∖	
	*Palmaria palmata* (Dulse), DW%	0.84	0.98	0.20	0.62	1.16	0.73	1.19	0.20	∖	
	*Porphyra umbilicalis* (Nori), DW%	1.75	1.70	0.29	1.06	2.20	1.25	1.63	0.47	∖	
**Plant**	Pea, DW% of protein	2.5	2.7	0.3	2.3	5.7	3.7	4.7	1.6	∖	Gorissen et al. (2018)
	Hemp, DW% of protein	1.3	1.3	1.0	1.0	2.6	1.8	1.4	1.1	∖	
	Soy, DW% of protein	2.3	2.2	0.3	1.9	5.0	3.2	3.4	1.5	∖	
	Chickpea, DW% of protein	3.0	4.6	1.1	4.8	8.5	5.3	7.0	3.2	∖	Rachwa‐Rosiak et al. (2015)
**Insect**	Bombay locust, DW%	1.21	2.65	0.38	1.48	2.91	0.94	1.71	0.76	0.23	Köhler et al. (2019)
	Scarab beetle, DW%	1.07	1.87	0.45	1.28	2.12	0.92	1.55	0.73	0.47	
	House cricket, DW%	1.20	2.01	0.44	1.09	2.34	1.03	1.73	0.69	0.29	
	Mulberry silkworm, DW%	1.10	1.36	0.77	0.95	1.70	1.15	1.69	0.68	0.34	
	Lesser mealworm, DW%	2.41	3.80	0.88	3.10	4.16	2.63	3.76	2.04	0.69	Malla et al. (2022)
	Yellow mealworm, DW%	1.94	3.30	0.60	2.63	3.68	1.75	2.45	1.41	0.55	
	Black soldier fly, DW%	1.65	2.70	0.66	2.23	2.98	1.67	2.16	1.14	0.62	

**TABLE 2:** Algae protein extraction methods: Conditions and effect on protein yield and quality for different algae.

Extraction method		Parameters	Protein yield	Protein quality and functionality	Algae	References
**Physical method**	Homogenization	5% protein concentrate, 50 MPa, and 100 MPa; cell disruption rate and energy consumption	Along with the pressure and passing times	Improve thermal gelation	*Arthrospira platensis*	Carullo et al. (2018), Geada et al. (2021), Shkolnikov et al. (2021)
	Bead milling	pH 6.5, 0.6 kWh/kg DW; be aware of heating and energy consumption	50.4% (DW) in the extract	High surface activity; excellent gelation	*Tetraselmis suecica*	Geada et al. (2021)
	High shearing	20,000 rpm, 96 kJ/kg_SUSP_; high efficiency	25.8% (w/w) of total proteins	Improved recover yields of intracellular compounds, including pigments; partial fragmentation of the trichomes	*A. platensis*	Carullo et al. (2021), Geada et al. (2021)
	Sonication	0%–100% power, 35–130 kHz; limited application scale, high energy consumption	25.3%–76.6%, dependent on pH; maximum 82.1%, with protease	High level of umami‐taste free amino acids; 50% flavored; free radical reaction	*Chlorella vulgaris*	Geada et al. (2021), Hildebrand et al. (2020)
	Pulsed electric field	Electric field strength 7.5–30 kV/cm each 5 s at batch mode; 20 kV/cm for 2 µs at continuous mode; mild cell disruption; energy input not relating to protein release; not competitive with mechanical method	Max 13% in *Neochloris oleoabundans* at batch mode; 2.5%–3.5% in *C. vulgaris* and 1.9%–2.5% in *N. oleoabundans* by continuous flow mode	∖	*C. vulgaris* and *N. oleoabundans*	Carullo et al. (2018), Lam et al. (2017)
	Micro‐fluidization	0–120 MPa	12% at 120 MPa	Protein degraded; released intracellular protein; reduced molecular weight; increased 20% digestibility in vitro	*Chlorella pyrenoidosa*	Ke et al. (2023)
**Chemical method**	pH adjustment	Alkalization	98% protein in pH 12; 71% protein in pH 7	lower functionalities than in pH7	*C. vulgaris*	Ursu et al. (2014)
	Isoelectric precipitation	Protein solubilization at pH 11, protein precipitation at pH 4.2 (with the aid of high‐speed homogenizer)	83.9 ± 1.7 wt% protein in protein concentrate; 91.3 ± 1.2 wt% protein in protein isolate	Increased solubility and foaming ability; resistant to thermal denaturation	*Spirulina* sp.	Pereira et al. (2018)
	Osmotic shock	20 g algae suspended in 1 L ultrapure water at 4°C	35.2%; effective extraction	Taurine (43% in *Fucus vesiculosus*) is a sulfonic acid but similar to amino acid; Aspartic acid (10.9%–12.1%) and glutamic acid (12.1%–12.3%) are the predominant amino acids in the other algae	*Fucus vesiculosus, Alaria esculenta, Palmaria palmata*, and *Chondrus crispus. F. vesiculosus*	O' Connor et al. (2020)
	Alcohol treatment	Methanol, solid to liquid ratio 20 g/mL; homogenization 120 MPa; washed with ethanol (∼20 g/100 mL)	25.8%	Increased whiteness; higher level of protein denaturation; reduced water solubility; decreased alanine and proline; higher digestibility; increased oil holding capacity and water holding capacity; higher foaming stability; higher level of chewiness, hardness, and gumminess	*C. pyrenoidosa*	Yang et al. (2024)
**Biological method**	Cellulase	*Macrocystis pyrifera* (18 h, 1/10 enzyme/seaweed ratio); *Chondracanthus chamissoi* (12 h, 1/10 enzyme/seaweed ratio)	*M. pyrifera* (74.6%) and *C. chamissoi* (36.1%)	Antioxidant activity (*M. pyrifera* 83 µmol/TE g, *C. chamissoi* 35 µmol/TE g); antihypertensive activity *(M. pyrifera)*	*M. pyrifera* and *C. chamissoi*	Vásquez et al. (2019)
	Xylanase	37 U were added to 300 g of frozen *P. palmata* (2 mg pure enzyme)	54.9% (conversion factor 6.25)	Enhanced amino acid content	*P. palmata*	Bjarnadóttir et al. (2018)

## 4 Functional Properties of Algae Proteins

**FIGURE 3:**
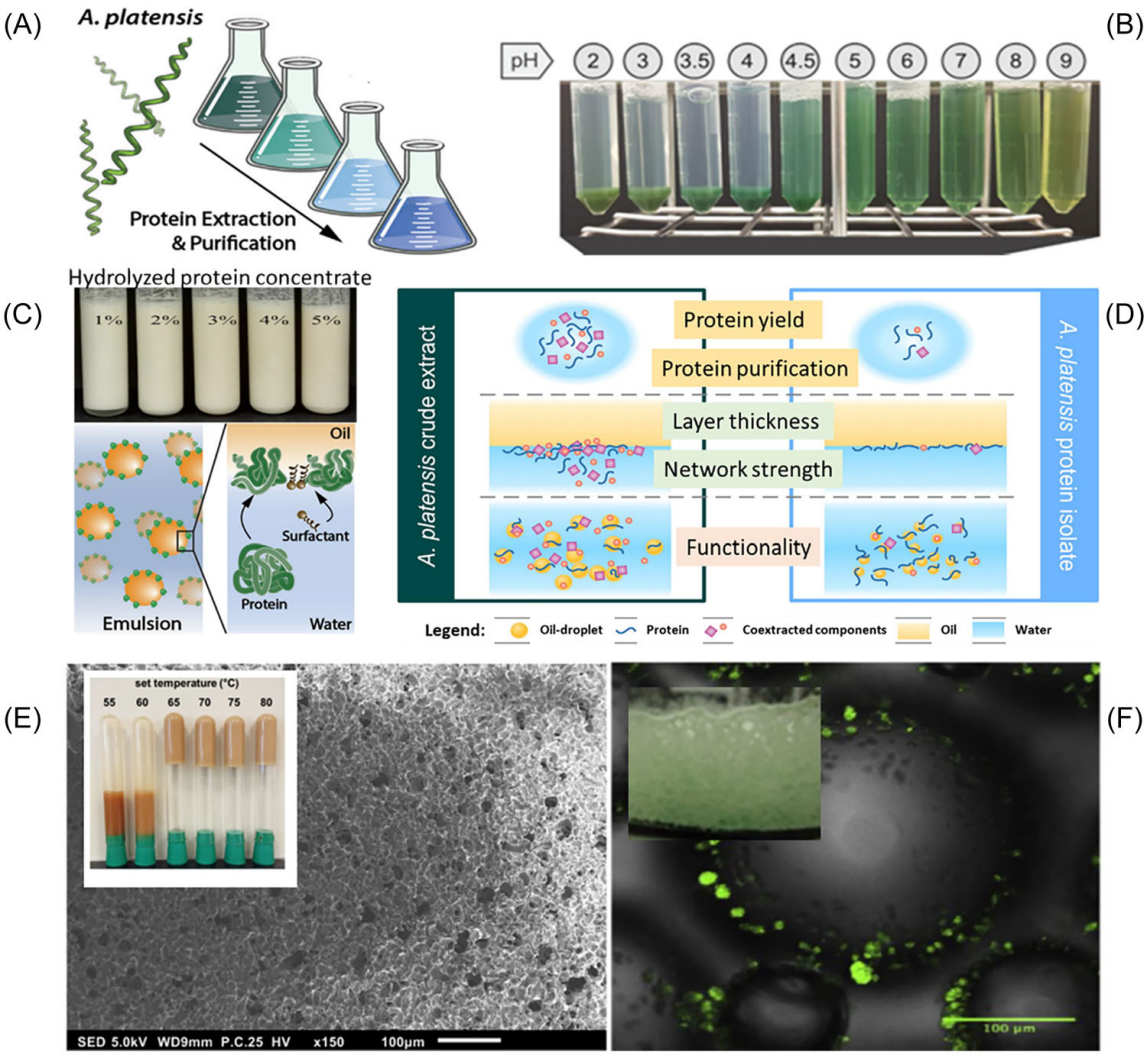
Functional properties of algal protein from extraction to solution and use in emulsification, gelation, and foaming: influence of process and conditions of use. Influence of extraction and purification on color (A); influence of pH on solubility (B); mechanism of emulsion stabilization, influence of algae protein concentration (C) and purity (D); algae protein gelation regulated by temperature, microstructure of gel network observed by scanning electron microscopy (E); foam stabilization by algae, interfacial behaviors of algae observed by confocal laser scanning microscopy (F). *Source*: (A) Adapted from Böcker et al. (2021). (B) Adapted from Böcker et al. (2021). (C) Adapted from Dai et al. (2020b) and Böcker et al. (2021). (D) Adapted from Böcker et al. (2021). (E) Adapted from Grossmann et al. (2019). (F) Adapted from Amagliani and Schmitt (2017) and Buchmann et al. (2019).

The next generation of plant‐based foods will likely face challenges related to quantity, diversity, cost, quality, and innovative processing. These challenges are heavily dependent on the structure‐function relationship and nutritional profiles of proteins in product development (McClements and Grossmann 2023). The functional properties of proteins are determined by their structure and assembly state, which are influenced by environmental factors and processing techniques (Figure 3), also known as techno‐functional properties (Buchmann et al. 2019; Waghmare et al. 2016). Gentle processing methods are preferred for protein extraction in food development to preserve protein functionality. Practically, harsh extraction procedures, whether physical or chemical, can significantly alter the hierarchical structures of algae proteins, thereby affecting their functional properties. For example, chemical methods that disrupt protein structures can enhance emulsification abilities. Both the initial protein production process and subsequent post‐processing steps can significantly modify protein functionality. For instance, sonication facilitates the formation of protein aggregates through free sulfhydryl bonds and surface hydrophobic groups (Dabbour et al. 2024). To gain a comprehensive understanding of algae protein production and its impact on protein quality, Waghmare et al. (2016) analyzed *Chlorella pyrenoidosa* as a model algae. The effects of various factors, such as solvent type, ammonium sulfate concentration, solid load, pH, incubation time, slurry‐to‐butanol ratio, and enzymatic treatment, on protein yield were investigated. They also examined how these factors influence protein characteristics, including composition, dried mass color, water and oil holding capacity, foaming capacity and stability, and thermal properties (Waghmare et al. 2016). Subsequently, it is of great importance to elucidate the relationship between protein structure and processing methods.

### 4.1 Solubility

#### 4.1.1 Mechanisms of Protein Solubility

Protein solubility is a crucial determinant of both the functionality and techno‐functional properties of proteins (Schwenzfeier et al. 2011). Low protein solubility can adversely affect the functionality of proteins, leading to reduced performance in various applications. Grossmann and McClements (2022) provided a comprehensive analysis of how environmental conditions, such as temperature, pH, ionic strength, and solvents, affect protein solubility by modifying the protein's surface properties and structural conformation (Figure 3B). For example, elevated temperatures can alter protein mobility and cause structural denaturation, leading to increased protein aggregation.

#### 4.1.2 Comparative Analysis With Other Proteins

The aggregation of algae polypeptides is notably influenced by the pH of solution, as evidenced by Bernaerts et al. (2017), who observed increased aggregation at pH 6 when subjected to thermal treatment. This behavior underscores the significant role of solution conditions in determining protein aggregation. In particular, ionic strength and pH have profound effects on protein structure and solubility. Like many plant‐based proteins, the solubility of algae proteins is highly pH‐dependent (Schwenzfeier et al. 2013; Waghmare et al. 2016). Algae proteins generally exhibit high solubility within the pH range of 6–11, with the lowest solubility occurring around pH 4. For example, when the pH of *Nannochloropsis oculata* protein was adjusted, the solubility plateaued between pH 5.5 and 8.5. At alkaline pH levels, the solubility of algal proteins averaged around 51%, whereas at pH levels lower than 5.5, solubility dropped below 9%. Therefore, pH levels of 7 and 10 are often considered optimal for maximizing protein solubility. Cavonius et al. (2015) optimized the pH shifting strategy for protein recovery, determining that precipitation at pH 3 while alkalization to pH 7 or pH 10 was optimal when balancing protein solubility, pellet sedimentation, and acid addition. *Arthrospira platensis* proteins displayed a U‐shaped solubility curve, with the lowest solubility (6.2% w/w) at pH 3 and the highest (59.6% w/w) at pH 10, reflecting its isoelectric point (pI) (Benelhadj et al. 2016). Studies on *Chlorella protothecoides* found that water‐soluble proteins had over 84% solubility across a broad pH range (2–12), with the minimum solubility observed at pH 2. Conversely, the water‐insoluble protein fraction showed low solubility over the entire pH range, without a distinct pI, with a maximum solubility 26.9% ± 2.8% observed at pH 12 (Grossmann et al. 2019). Schwenzfeier et al. (2011) reported that *Tetraselmis* sp. protein isolates achieved 64% w/w protein content, with the isolates being colorless and independent of ion conditions, unlike plant proteins (Schwenzfeier et al. 2011). In summary, pH shifting is a potent method for modulating the solubility of algae proteins. For instance, *Kappaphycus alvarezii* protein concentrates showed pH and salt‐dependent solubility, with the lowest nitrogen solubility (33.72% ± 1.23%) at pH 4, attributed to the destabilizing effect of the pI (Suresh Kumar et al. 2014). Additionally, *Chlorella sorokiniana* dry powder, when dispersed at an alkaline pH of 8 post‐homogenization, exhibited enhanced protein solubility (Li et al. 2024).

### 4.2 Emulsifying Ability

#### 4.2.1 Mechanisms of Protein Emulsifying Ability

The emulsifying properties of algal proteins are intrinsically linked to their structural characteristics. The extraction process can enhance these emulsification properties simultaneously. For instance, protein extracted from brown seaweed demonstrated a water‐holding capacity of 10.27 ± 0.09 g H_2_O/g and an oil‐holding capacity of 8.1 ± 0.07 g oil/g (Garcia‐Vaquero et al. 2017). Despite this, the algal proteins with clearly known structure, even after filtration, can complicate their interfacial behavior. Algae can stabilize Pickering emulsions by acting as solid particles within the emulsion (Ebert et al. 2019; Mishra et al. 2020; Teuling et al. 2019).

Interestingly, purified algal extracts have been shown to improve emulsifying ability, but crude algal extracts also perform well in stabilizing emulsions. For example, emulsions prepared with crude extracts (0.5 w/w%) had a mean droplet size (d43) of 5.0 ± 0.1 µm, whereas those made with purified algae protein at the same dry mass concentration had a larger droplet size of 10.6 ± 0.2 µm (Figure 3A–C) (Böcker et al. 2021). However, Pickering emulsions stabilized with insoluble algal proteins were found to be unstable due to flocculation during storage (Dai et al. 2020a, 2020b).

#### 4.2.2 Comparative Analysis With Other Proteins

Soluble protein isolates from *Tetraselmis* sp. exhibited superior emulsifying performance and stability compared to whey protein isolate at pH 5, attributed to the co‐absorption of polysaccharides. This high emulsifying capacity is also due to the elevated protein content and stability (Schwenzfeier et al. 2013). Long‐term stability of algal protein isolates is crucial, with *C. sorokiniana* proteins maintaining a monomodal droplet size distribution (d43 = 232 ± 22 nm) and stability over 7 days. In contrast, *Phaeodactylum tricornutum* proteins, despite higher protein content (3.7 wt%), showed less stability, with a droplet size of d43 = 334 ± 12 nm, but better performance under storage or salt conditions. However, *C. sorokiniana* protein demonstrated superior stability compared to *P. tricornutum* across pH treatments (Ebert et al. 2019).

The solution characteristics of algal proteins during rehydration are closely linked to their emulsifying ability. For example, *A. platensis* protein isolate, with an oil absorption capacity of 252.7 ± 9.9 g, exhibited lower emulsifying ability (44.1% ± 0.9%) at its pI, which increased when deviated from this pH (Benelhadj et al. 2016). *Haematococcus pluvialis* protein showed significant emulsification potential both at its native pH and when adjusted to neutral pH (Ba et al. 2016).

The protein structure affects emulsifying ability through its behavior at interfaces. At a protein concentration of 6 g/L, algae proteins exhibited emulsifying capabilities similar to, though slightly lower than, sodium caseinate, which is known for its high emulsification capacity due to its unfolded and dissociated structure (Ba et al. 2016). This suggests that improving the emulsifying ability of algal proteins might be achievable by disrupting and stretching their structures. This insight parallels findings in plant proteins, where gentle chemical or enzymatic modifications enhance surface activity by exposing hydrophobic groups (Wojciechowski 2022). Plant proteins typically have a high β‐sheet and low α‐helix content (Le Roux 2019). Additionally, acid‐hydrolyzed insoluble microalgae proteins from *C. protothecoides* were effective in stabilizing emulsions against salt changes, indicating that electrostatic forces are not the sole factor in flocculation. Emulsions prepared at extreme pH conditions (2–3 or 8–9) were more stable compared to those at the pI (Dai et al. 2020a). The polarity of proteins can shift from polar to nonpolar at the oil–water interface due to protein unfolding (McClements 2023). The emulsifying capacity of proteins from various photosynthetic unicellular organisms depends critically on the protein concentration that covers the droplet surface (Teuling et al. 2019). Dai et al. (2021) investigated the effectiveness of insoluble algal proteins and their hydrolysates in stabilizing oil‐rich emulsions containing over 50% oil. The hydrolysis was conducted using 0.5 mol/L HCl at temperatures of 65°C or 85°C for 4 h. Compared to those hydrolyzed at 65°C, the hydrolysate obtained at 85°C, characterized by its gel‐like structure, demonstrated a superior ability to emulsify and stabilize emulsions more concentrated in fats (Dai et al. 2021). These findings underscore the need for a comprehensive database on the surface activity of algal proteins to enhance our understanding and application of these proteins in various emulsification processes.

### 4.3 Foaming Ability

#### 4.3.1 Mechanisms of Protein Foaming Ability

The foaming properties of proteins are primarily due to their ability to migrate to the air‐water interface, reducing surface tension and stabilizing the foam to prevent coagulation (Waghmare et al. 2016). Proteins in food can function as either foam‐forming or foam‐stabilizing agents. For example, the foaming capacity of egg white protein is attributed to the combined effects of lysozyme, ovomucin, and ovalbumin. Similarly, algal proteins, being a mixture of various proteins, may exhibit comparable foam formation mechanisms (Murray 2020).

Foam‐stabilizing molecules can be categorized by their size, with proteins as larger molecules attaching to the foam interface (Figure 3F). The flexibility of these proteins affects the formation of the interfacial film. A strong film covering the interface enhances foam stability (Amagliani et al. 2021).

#### 4.3.2 Comparative Analysis With Other Proteins

High flexibility in algae proteins can potentially be achieved by disintegrating their structure. For instance, globular plant proteins require greater flexibility for effective interfacial absorption (Amagliani and Schmitt 2017). Compared to whey protein and egg white albumin, the foaming ability of soluble algae protein is superior within a pH range of 5–7. Protein adsorption increases at high ionic strength and decreases near the isoelectric pH (Schwenzfeier et al. 2013). Acid‐hydrolyzed algae protein at 85°C enhances foam formation, achieving a half‐life of 2–3 h at concentrations as low as 0.1% and producing the largest foam volume with the smallest bubble diameter. At 5% protein concentration, the foam's half‐life extends to 5 h (Dai, Shivananda et al. 2020). Residues remaining after protein extraction, such as polysaccharides, can also affect the solubility and gelation ability of algae proteins (Schwenzfeier et al. 2014). The foaming ability of algae protein is pH‐dependent. For instance, *H. elongata* protein concentrate shows the lowest foaming ability at pH 2 (6.98% ± 0.16%) and the highest at pH 10 (71.52% ± 4.81%), with intermediate values at pH 6 (64.44% ± 2.22%) and pH 8 (55.56% ± 6.92%) (Garcia‐Vaquero et al. 2017).

### 4.4 Gelation Ability

#### 4.4.1 Mechanisms of Protein Gelation Ability

Gelation is a common feature in various foods, enhancing taste and mouthfeel. Examples of gelled foods include yogurt, emulsified sausage, pudding, jam, and soft cheese. Integrating algae into these gelled foods as a nutrient enhancer or creating novel gelled algae‐based foods is meaningful. Gelation can be achieved through heat treatment, enzymatic action, or acidification. The protein gelation like whey is well‐known, whereas the gelation of algal proteins is less explored. Food gel formation can be classified into physical methods (heat, pressure), chemical methods (acid, ions), and biological methods (microorganisms, enzymes). Algal proteins require unfolding to change their original structure. Amino acids like histidine, lysine, and arginine exhibit pH‐dependent effects on protein gelation (Wang et al. 2020).

The formation of an algal protein network involves both non‐covalent and covalent bonds, bridging proteins or proteins with other components. Dominant forces in gel structure formation include hydrophobic interactions, hydrogen bonds, and disulfide bridges formed by cysteine residues.

#### 4.4.2 Comparative Analysis With Other Proteins

Aggregates may embed into the gel matrix, contributing to the unique gelation behavior of algal proteins. For instance, *Spirulina* protein denatures at temperatures above 60°C, forming initial crosslinking interactions related to the degree of aggregation. Although intermolecular disulfide bonds are not essential for forming the gel structure, they can strengthen it. Compared to other food proteins, the critical gelling concentration of *Spirulina* protein isolate is relatively low, which is around 1.5% wt (Chronakis 2001). Grossmann et al. (2019) demonstrated that algae protein extracted from *C. sorokiniana* could form a stable gel structure with as little as 9.9 g protein per 100 mL at 61°C (Figure 3E). The gelation of algae protein is also influenced by environmental conditions. The gel structure is adversely affected by intensified ionic conditions and pH deviations from the native value (Grossmann et al. 2019). A flexible application of gelation properties is in 3D printing technology, which can tailor nutritional food for people at different ages. Notably, high dry mass *Spirulina* slurry mixed with vegetable oil was found to be printable as a viscoelastic emulsion gel with a plastic‐like response (Feng et al. 2023).

## 5 Applications and Technological Barriers of Proteins

The traditional consumption of algae often involves direct incorporation into dishes or cuisines, such as using kelp in Asian cooking. However, broadening the methods of algae consumption is essential for advancing its application in the food industry. For instance, recent innovations have incorporated algae into noodles to enhance their protein content (Rodríguez De Marco et al. 2014). The successful application of algae protein largely depends on advances in protein processing technology. Algal protein has the potential to substitute animal‐based proteins in various major food categories, including meat, milk, and eggs (Table 3). In the context of plant‐based meat alternatives, algae are typically used in powdered form and processed through high‐moisture or low‐moisture extrusion. Similarly, for milk analogs, a powder matrix is crucial for easy handling and storage but requires adapted formulation and good rehydration properties. However, powder form may not be as critical for egg alternatives, especially at market for customers.

**TABLE 3:** Algae as a protein replacer in meat product or dairy product (L*: lightness; a*: red/green value; b*: blue/yellow value in CIELAB color chart).

Food		Algae species	Protein and amino acids	Food quality	References
Meat	Sausage	*Spirulina* and *Chlorella*	Higher level of glutamic acid, aspartic acid, lysine, and leucine	Higher pH than non‐algae samples; lower a*; higher ash level; good water holding capacity >4.30 g/100 g; deteriorated texture	Marti‐Quijal et al. (2019)
	Chicken breast formulation	*Spirulina* and *Chlorella*	Higher level of total amino acids and glutamic acid in *Spirulina*; higher level of essential amino acids; good alternative protein in meat	Higher pH; lower L*and a*, higher b*; decreased hardness, lower elasticity; deteriorated cohesiveness, gumminess, and chewiness	Marti‐Quijal et al. (2018)
	Patties	*Spirulina* and *Chlorella*	High in total amino acids and essential amino acids; good source of sulfur containing amino acids	Lower a* and b*; slightly higher pH; texture not affected; no obvious taste change	Žugčić et al. (2018)
	Extruding meat analog	*Auxenochlorella protothecoides* and soy protein concentrate	Undisrupted microalgae cells limit intracellular protein; hinder fibrous protein cross‐linking	Decreasing cutting strength; reduced harness; resemble to chicken	Caporgno et al. (2020)
	Extruding meat analog	*Spirulina* and soy protein concentrate	Suitable for partial replacement of soy protein	Intensified chicken flavor; darker product; fibrous, elastic, firm and layered product with 30%–50% *Spirulina*; juicy and soft mouthfeel; affect springiness	Grahl et al. (2018)
Dairy	Yoghurt	*Isochrysis galbana*	Higher protein content	Rich in DHA; innovative green tonality	Matos et al. (2021)
	Liquid milk	*Ascophyllum nodosum*	Protein‐polyphenol interaction	Stable solution; DPPH and ferrous‐ion‐chelating activities; increased a*and b*, decreased L*; negative sensory, fishy taste	O'Sullivan et al. (2014)
	Cheese	*Spirulina platensis*	Increased protein content	less intense odor and taste	Bosnea et al. (2021)

In addition to liquid forms, algae products or derivatives are frequently used in powdered form (Su et al. 2023). Drying processes are employed to remove water from algae, reducing water activity and facilitating long‐term storage. However, intensive drying methods can compromise the nutritional value of the algae. For example, sun drying led to the disintegration of antioxidant components like vitamin C (Khaled et al. 2024). In addition, the properties of the powdered matrix are influenced by the drying technique used. Spray drying is commonly employed for producing food powders due to its ability to provide consistent quality and support continuous production. Factors such as feed viscosity, droplet size, and drying temperature can affect the properties of the powder matrix and the bioactivity of the microalgae (Vilatte et al. 2023). Freeze drying can also be considered for algae powder production, but reports from some companies indicate that freeze‐drying can reduce the level of biomolecules in the biomass by up to 50%. A promising alternative is electrostatic spray drying, allowing drying under lower air temperature (Jayaprakash et al. 2023).

### 5.1 Meat Analog

Between 1961 and 2014, global average meat consumption increased more rapidly than the population growth, rising from 20 to 43 kg per person per year (Ritchie et al. 2017). Red meat has been associated with adverse health effects, including cardiovascular disease and cancer (Aykan 2015). In response to these concerns, there has been a surge in the development of plant‐based meat analogs, driven by their potential benefits for the environment, animal welfare, and nutrition (Ryu et al. 2023).

**FIGURE 4:**
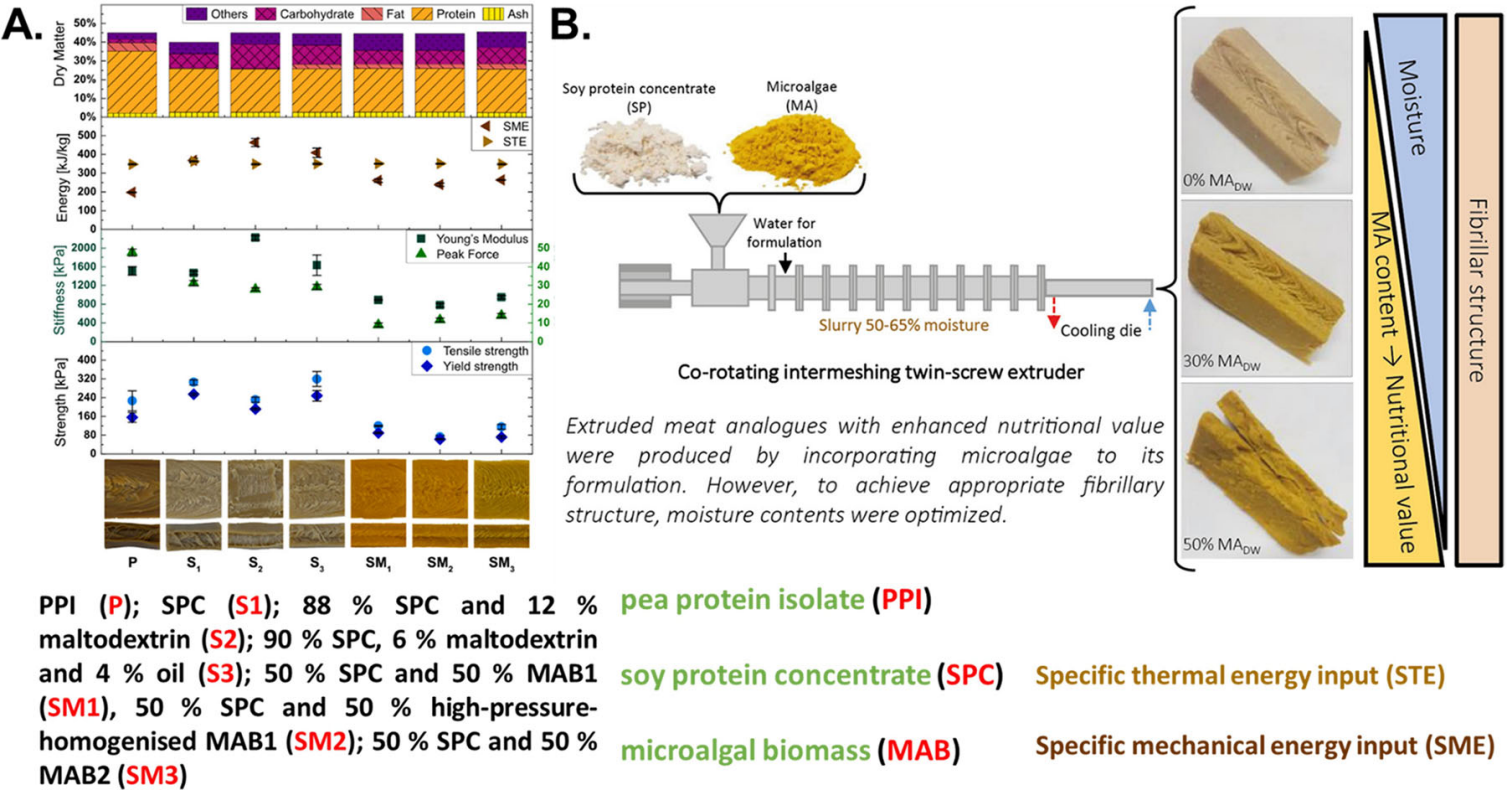
Meat analog by high moisture extrusion: Influence of formulation on physico‐chemical properties and fibrillary structure for soy, pea, and algae protein extrudates (Sägesser et al. 2024) (A); soy protein and algae extrudate (Caporgno, et al. 2020) (B).

Meat analogs from algae can generally be categorized into two types. The first category involves using algal protein as a primary ingredient in high‐moisture extrusion processes to create fibrous meat substitutes (Figure 4). This method replaces animal proteins entirely with plant proteins or algae biomass. However, challenges in this approach include replicating the appearance and texture of traditional meat. For instance, red‐colored algae can be used to mimic the color of meat because of its red color, which is similar to hemoglobin (Fu et al. 2021). Although high‐moisture extrusion of *Spirulina* can produce a fibrous structure, it may result in a darker color, a stronger earthy flavor, and reduced elasticity and firmness (Grahl et al. 2018). Combining algae protein with plant proteins can help refine the texture of meat analogs. Research has shown that the microalgal cell wall and fat can hinder the formation of a fibrous structure, with the cell wall impeding protein interactions. A 30% addition of microalgae at 60% moisture content has been found effective in high‐moisture extrusion processes after optimization (Figure 4B) (Caporgno et al. 2020). Recent studies on high‐moisture extrusion combining algae with pea protein have highlighted that microalgal proteins with smaller molecular weights, higher solubility, and fewer hydrophobic sidechains may be less effective for forming a fibrous structure, underscoring the importance of protein composition, properties, and the electrolyte environment (Figure 4A) (Sägesser et al. 2024). Additionally, replicating the fat content in meat analogs is crucial. Companies like Profilet are focusing on algae‐based alternatives to fish, indicating a growing market and continued innovation in meat analogs.

**FIGURE 5:**
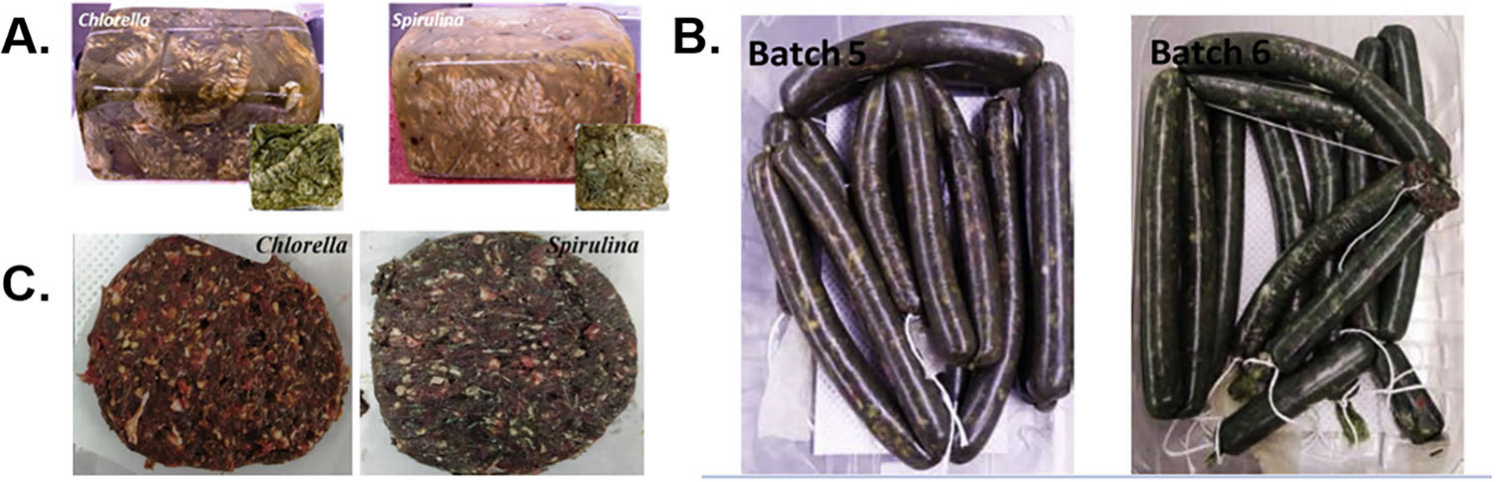
Meat replaced by algae: turkey breast formulation (Marti‐Quijal et al. 2018) (A), fresh pork sausages (Marti‐Quijal et al. 2019) (B), and beef patties (Žugčić et al. 2018) (C).

The second category of meat analogs incorporates algae as an additive to traditional meat products (Žugčić et al. 2018). Algae can be included in various meat products, such as sausages, burgers, and patties (Figure 5) (de Medeiros et al. 2021; Marti‐Quijal et al. 2019). Different algae formulations affect the composition and characteristics of the final product. For example, chicken roti with 5.82 g/100 g *Spirulina* has a higher fat content compared to a sample with 5.04 g/100 g soy, which was ascribed to the lipid in algae. Generally, substituting plant proteins with algae can reduce the hardness, elasticity, and chewiness of meat products (Marti‐Quijal et al. 2019; Marti‐Quijal et al. 2018; Parniakov et al. 2018). Additionally, whole algal biomass can serve as a natural salt replacer in meat products, offering a potential reduction in sodium content (Espinosa‐Ramírez et al. 2023).

### 5.2 Dairy Analog

**FIGURE 6:**
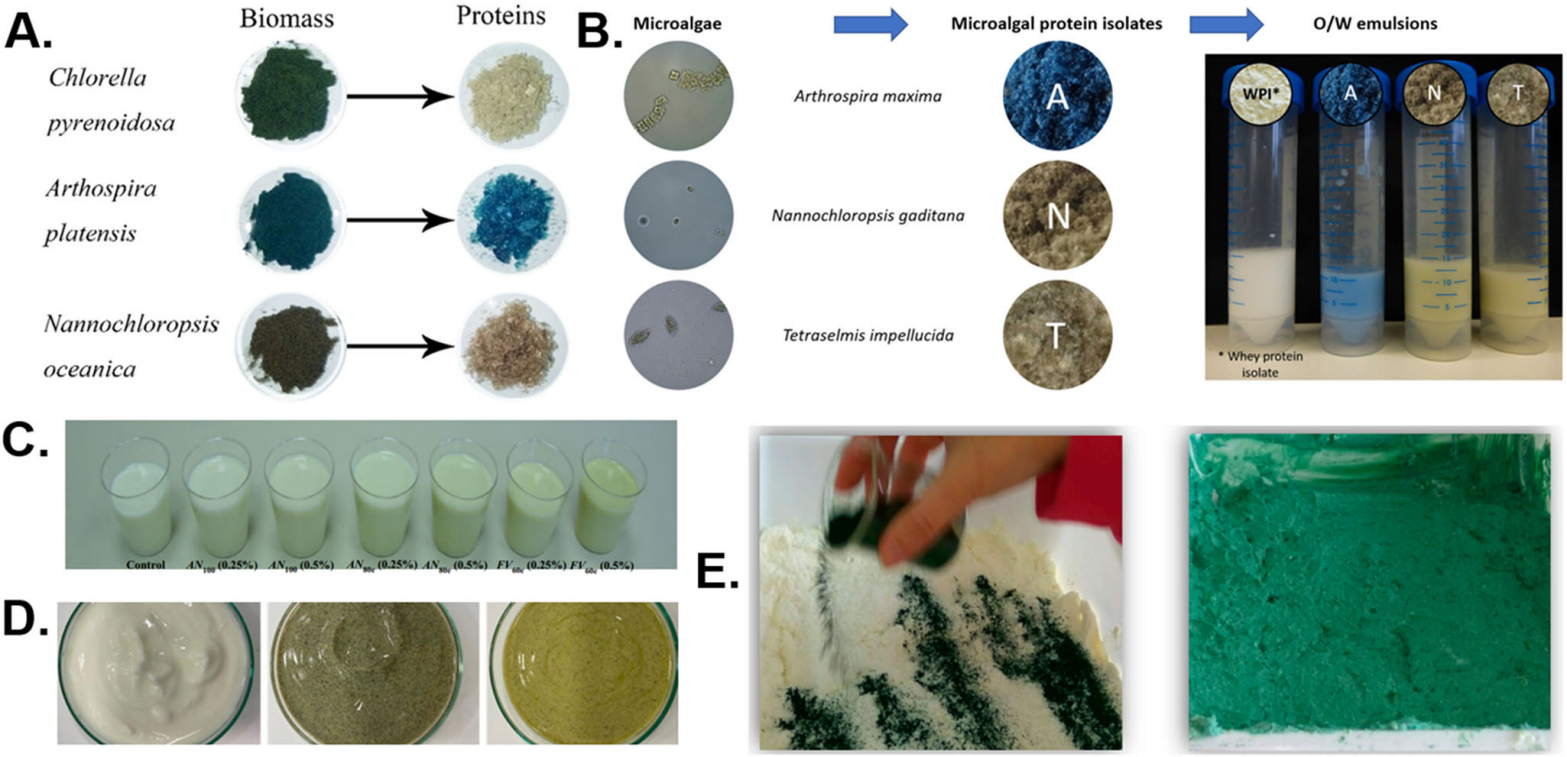
Algae protein extracted from biomass (A); Microalgae processed into microalgal protein isolates followed by emulsions (B); algae added into dairy milk (C), in yoghurt (D), in cheese (E). *Source*: (A) Adapted from Chen et al. (2019). (B) Adapted from Teuling et al. (2019). (C) Adapted from O'Sullivan et al. (2014). (D) Adapted from Matos et al. (2021). (E) Adapted from Bosnea et al. (2021).

Global milk consumption increased significantly from 2017, reaching 828 million tons, with 83% of this total attributed to bovine milk (Górska‐Warsewicz et al. 2019). A notable advancement in the development of milk alternatives has been made by Sophie's Bionutrients, a Singaporean company that claims to have produced the world's first microalgae‐based milk. Additionally, incorporating seaweed extract into milk has been reported to enhance its antioxidant properties, improve shelf life, and fortify both its nutritional profile and texture (Figure 6). Microalgae have also been integrated into fermented dairy products such as cheese and yogurt to offer additional health benefits (Figure 6C–E) (Beheshtipour et al. 2013; Hernández et al. 2022; O'Sullivan et al. 2014; Roohinejad et al. 2017). In the dairy industry, proteins are typically isolated from raw milk and tailored for specific functionalities. However, algae proteins require an initial extraction process (Figure 6A,B). To use algae, the formulation and design of milk analogs must carefully address emulsion stability and overall nutritional content.

The physical and functional stability of milk analogs against environmental factors involves complex considerations. Factors such as small particle size and the addition of thickening agents can enhance the physical stability of milk analogs. Extreme pH levels, temperature fluctuations, and excessive shearing or shaking can lead to flocculation. Common defects in milk analogs include gravitational separation, creaming, sedimentation, flocculation, and coalescence (McClements 2020). For high‐demand products like infant formula, which require stringent food safety and nutritional standards, alternative proteins are being explored (Le Roux 2019). Le Roux et al. (2020) found that pea and faba proteins resulted in reduced emulsion stability compared to real milk, exhibiting flecking upon powder rehydration. In contrast, soluble fractions of algae proteins are recommended for developing alternative infant formulas. Fortunately, the remaining algae protein isolates are often rich in charged polysaccharides, which contribute to improved emulsion stability (Teuling et al. 2019). The structure of algae proteins also affects their emulsifying abilities, with disrupted proteins showing better emulsification properties compared to less soluble fractions (Dai et al. 2020a, 2020b). Algae proteins are typically dark green, but alkaline conditions can protect chlorophyll while acidic conditions can degrade it, depending on the desired color (Cavonius et al. 2015). Decolorization can also be achieved through acidification sediment, as demonstrated with *Tetraselmis* sp. at pH 3.5 (Schwenzfeier et al. 2011). The shelf life of milk analogs depends on the stability of the product during storage. Seaweed extracts have been shown to provide DPPH radical scavenging activity, enhancing emulsion oxidative stability (Figure 6C) (O'Sullivan et al. 2014). However, research on the shelf life of algae milk analogs remains limited due to the nascent stage of alternative protein development.

Despite their unique nutritional profiles, algae‐based milk analogs often require supplementation to match the critical nutrients found in traditional milk. Comparative studies show that bovine milk excels as a natural calcium carrier, a nutrient often deficient in dairy analogs (Tangyu et al. 2019). Therefore, plant‐based milk alternatives should be fortified with calcium. Zhou et al. (2021) found that while calcium fortification is crucial, excessive levels can negatively impact vitamin D bioaccessibility, highlighting the need for careful nutrient formulation. Additionally, incorporating lactic acid bacteria into dairy analogs can reduce anti‐nutrient factors (Erem and Kilic‐Akyilmaz 2024).

In dairy products, the food matrix can be either liquid or solid. Semi‐solid dairy products include yogurt, whereas solid dairy typically refers to powder‐based foods. Gelation in real milk is driven by the cross‐linking of casein micelles. The addition of *Nannochloropsis salina* can hinder this gelation process due to ruptured cells and insoluble debris. Enhancing the solubility of algal debris can improve gel structure (Muñoz‐Tebar et al. 2022). Replacing casein with algae or algae combined with plant proteins results in a different gelation process, often requiring thermal treatment. For instance, a 9.9 g/100 mL algae sample could form a gel structure when heated to 61°C. Small algae protein‐rich particles formed by high shearing can improve gel structure when combined with soy protein isolate (Grossmann et al. 2019; Wang et al. 2023).

### 5.3 Egg Analog

Alongside meat and milk analogs, egg analogs occupy a significant share of the alternative food market. The future of this market is promising, with expectations of high production volumes, targeted marketing, and clear regulations to drive growth. Consumers are increasingly choosing vegan eggs for their cholesterol‐free and clean label benefits (Boukid and Gagaoua 2022). High‐quality algae protein contributes to the appeal of vegan eggs. However, processing algae protein to effectively substitute real eggs presents some challenges. Besides the difficulty in achieving the desired color for egg analogs, identifying key volatile compounds in algae remains a significant hurdle (Urlass et al. 2023). Currently, egg analogs are primarily available in liquid form, sterilized by pasteurization, and requiring further cooking to form a gel‐like structure. This liquid form must mimic the gelation process of real eggs, which is heat‐induced and irreversible upon cooling. Real eggs contain ovotransferrin and ovalbumin, which exhibit two distinct gelation peaks at 66°C and 81°C (Liu et al. 2020). The critical point for protein denaturation and gelation at 66°C must be replicated in egg analogs. So far, mung bean protein has shown promise in mimicking this gelation process, a development accelerated by the protein platform of JUST EGG, which combines science lab and kitchen lab to select protein sources. Additionally, plant‐based emulsion gels can be used to create egg yolk alternatives (Li et al. 2024). Inspired by plant‐based eggs, algae‐protein eggs could be developed on the basis of their gelation abilities. Research by Zhou et al. (2022) explored the potential of RuBisCo proteins, finding that despite a gelation peak around 66°C, RuBisCo proteins exhibited a single denaturation thermal peak and produced a more fragile gel. RuBisCo proteins are found not only in higher plants but also in algae, indicating significant potential for using algae‐derived proteins in egg analogs. But this requires experimental result to check the possibility of egg analog by RuBisCo proteins from algae.

## 6 Quantitative and Qualitative Algae Protein Production

Before being utilized in the food industry, algal protein is extracted from both edible macroalgae and microalgae. Typically, algae protein is consumed in the form of algal biomass, which contains proteins that are not fully purified. Alternatively, algal protein can be isolated from the biomass, providing more controlled functionality. The extraction process involves careful selection between chemical and physical methods for concentrating algal protein (Pereira et al. 2018). The general protocols for obtaining algal protein include fractionation and purification techniques such as membrane ultrafiltration and chromatographic methods, often combined with ion‐exchange and gel‐permeation chromatography (Ejike et al. 2017). Among some algae (*A. (Spirulina) maxima*, *N. gaditana*, *T. impellucida*, and *Scenedesmus dimorphus*), these methods generally yield protein isolates with a concentration of 62%–77% w/w in dry mass, resulting in a protein yield of 3%–9% w/w by mild isolation, irrespective of the protein sources and extraction methods used. Protein concentration processes depend significantly on protein solubility, which is influenced by pH and the solution environment. Efficient and rapid recovery of protein is crucial for novel applications in protein utilization (Teuling et al. 2017).

### 6.1 Cultivation and Production of Algae Biomass

The species of algae significantly impact the yield of protein after cultivation. For instance, *Spirulina* spp., *Chlorella* spp., and *Dunaliella salina* are frequently utilized due to their high protein content (Boukid et al. 2021). Algae are valuable for food production because they offer high protein levels, rapid growth rates, and adaptability to various water sources (Deepika et al. 2022). The scale of algae cultivation plays a critical role in determining the yield and applicability of algae. Small‐scale cultivation can substantially increase the cost of developing algae‐based food products. Consequently, scaling up cultivation operations can be advantageous for cost control. Additionally, algae absorb carbon from air or water to produce protein biomass, contributing to sustainable and carbon‐neutral food production (Ahmad and Ashraf 2023). After cultivation, algae can be dried using various methods, including freeze‐drying, hot‐air drying, convective drying, infrared drying, spray drying, and tray drying. Drying enhances the algae's storage stability, transportation efficiency, and suitability for processing within the food industry (de Farias Neves et al. 2019).

### 6.2 Protein Extraction and Enrichment

Extraction is the process of isolating and concentrating algal proteins from biomass (Figure 6A). Advances such as thermal treatment and high‐speed centrifugation have simplified and improved protein extraction methods (Wang et al. 2022). However, low protein recovery rates may result from complex and inefficient extraction processes during refinement. Therefore, a more sustainable and straightforward method for extracting components from algae cells is needed.

Recovering soluble proteins from microalgae remains challenging due to the need to balance algal biomass quality with energy consumption. The efficiency of protein extraction is influenced by factors such as biomass concentration, physicochemical conditions during cell disruption, and subsequent processing steps, including bead milling, centrifugation, and microfiltration. Optimizing these processes can reduce the cost of microalgal protein extraction and purification to approximately €0.35/g of protein (Liu et al. 2021). Minimal processing methods, such as cell disruption followed by centrifugation and lyophilization, have also been reported to be effective for extracting water‐soluble proteins (Grossmann et al. 2018).

Algae protein extraction can be achieved through physical, chemical, or biological methods (Table 2). The choice of method depends on the specific characteristics of the algae and the conditions used. Mechanical methods are particularly crucial for enhancing protein yield. For example, the effectiveness of various extraction techniques is ranked as follows: high‐pressure cell disruption > chemical treatment > ultrasonication > manual grinding. Algae with more delicate cell structures are more easily disrupted, with the following order of susceptibility: *H. pluvialis* < *N. oculata* < *Chlorella vulgaris* < *Porphyridium cruentum* ≤ *A. platensis* (Safi, Ursu, et al. 2014).

#### 6.2.1 Physical Methods

Physical methods are commonly employed to disrupt the protective structures of plant cells, including algal cell membranes, which inhibit protein release during extraction. Among these methods, homogenization has been shown to be more effective than enzymatic treatments for disrupting algal cells and enhancing protein extraction. However, the efficiency of protein recovery can be influenced by the type of filtration membrane used. Interestingly, large cut‐off membrane (1000 KDa) had more severe fouling compared to smaller pore membrane (300 KDa), which resulted from the readily blocking of the large pores by polysaccharides (Safi et al. 2017). For instance, algae concentration in inlet feed, such as for *Nannochloropsis* sp., can reach up to 25% (w/w). Interestingly, the effectiveness of homogenization is more dependent on the pressure applied rather than on the feed concentration (Yap et al. 2015). Microfluidization is another technique that generates high energy by fluids collision in a high speed, which breaks particles down to micro‐ and nanoscale sizes. This method improves digestion of algae by breaking down cell walls, with pressures of 120 MPa resulting in 98% cell breakage and a 12% protein extraction rate. Furthermore, this process yields proteins with smaller molecular weights (Ke et al. 2023). The extraction process significantly affects the surface activity of crude *A. platensis* powder, although pigments often remain in the biomass post‐extraction. Pulsed electric fields can mitigate negative impacts on the techno‐functional properties of protein isolates (Buchmann et al. 2019). For optimal solubility and functionality, membrane filtration is recommended over sedimentation (Bleakley and Hayes 2017). Bead milling is another effective method for cell disruption, particularly for robust algae species like *N. oculata* and *P. cruentum*. High‐pressure effects are analyzed using residence time distribution modeling to assess and optimize operating parameters, including stress intensities and stress numbers (Montalescot et al. 2015). Additionally, steam explosion is a cost‐effective method for achieving complete cell disruption, which reduces overall extraction costs (Lorente et al. 2017).

#### 6.2.2 Chemical Method

Chemical methods play a crucial role in modifying the protein structure during extraction to enhance concentration. One widely adopted approach is pH shifting on the basis of the pI of the algal protein. This method often involves acid extraction followed by alkaline extraction, which can achieve a combined recovery efficiency of up to 59%, outperforming the 57% recovery obtained through a combination of alkalization and ultrasound treatment. Alkalization is a critical component of effective combined extraction strategies (Kadam et al. 2017). In another study, algae protein was first solubilized under alkaline conditions (pH = 11) and then precipitated by acidification (pH = 4.2), reaching the pI with the aid of homogenization. This method resulted in protein concentrates and isolates with purity levels of 83.9% ± 1.7% w/w and 91.3% ± 1.2% w/w, respectively (Pereira et al. 2018). Bertsch et al. (2023) demonstrated that hydrochloric acid could effectively precipitate soluble algae protein. Sodium hydroxide is particularly effective in penetrating the cellulose microcrystalline structure and dissolving hemicelluloses protected by cellulose‐rich cell walls, as observed in species like *C. vulgaris* and *N. oculata* (Safi, Charton, et al. 2014). Temperature also influences extraction efficiency; for instance, solubilizing *Nannochloropsis* biomass at 60°C and pH 11, followed by recovery at pH 3.2, can yield protein levels ranging from 40.5% to 56.9%, depending on the degree of biomass defatting (Gerde et al. 2013).

#### 6.2.3 Biological Method

Biological methods rely on enzymatic activity to facilitate the extraction of protein from algae. Enzymes play a crucial role in degrading the algal cell wall, thereby releasing protein components for further processing. Research indicates that enzyme‐assisted disruption of the algae cell wall, often combined with other extraction techniques, enhances protein recovery. For example, after optimizing enzymatic treatment and incorporating three‐phase partitioning, protein concentration achieved 78.1% w/w (Waghmare et al. 2016). Additionally, specific enzymes, such as xylanase, can significantly improve the yield and amino acid profile of algal proteins. For instance, xylanase treatment increased the extraction yield of protein from red seaweed (*Palmaria palmata*) to 80% after enzymatic pre‐treatment (Bjarnadóttir et al. 2018). Cellulase could effectively degrade fibrillar skeleton whose matrix is of sulfated galactans, which reached a 36.1% protein yield in *Chondracanthus chamissoi* (Vásquez et al. 2019).

#### 6.2.4 Integrated Methods

Single‐method approaches often fall short in effectively releasing protein from algal cells. Consequently, integrating multiple methods can significantly enhance protein extraction efficiency. For instance, combining alkaline treatment with mechanical methods can produce a synergistic effect, as demonstrated by Safi (2013). The integration of mechanical treatment and pH shifting has been shown to increase the yield of water‐soluble biomolecules in *H. pluvialis* (Ba et al. 2016). High‐pressure homogenization, in particular, has proven highly effective, releasing approximately 49% of proteins compared to 35% achieved through enzyme treatment alone. Additionally, integrating filtration with enzymatic processing has been found to enhance protein recovery, achieving a protein concentration of 24.8% (Safi et al. 2017). A multi‐step approach can also significantly improve protein quality. For example, the protein quality of *Tetraselmis* spp. was greatly enhanced through a series of processes, including bead milling, centrifugation, ion exchange chromatography, and decolorization, resulting in a protein level of 64% w/w in soluble isolates (Schwenzfeier et al. 2011). Overall, combined methodologies, even within the same category, tend to yield higher protein extraction levels. Chemical and biological methods often soften the algal structure, whereas intensive physical methods further maximize protein extraction from algal cells.

### 6.3 Protein Degradation and Bioactivities

The degradation of algae protein can be achieved through physical, chemical, or biological methods, each altering the protein's structure. This degradation can be a prerequisite for protein digestion and amino acid absorption. The effectiveness of algae protein digestion is closely related to the extent of its structural denaturation and degradation. Additionally, the bioactivity of algae protein is influenced by its degraded state. The bioactivities of peptides include anti‐inflammatory, antioxidant, antidiabetic, antiobesity, and so forth. To evaluate the bioactivity of algae protein, several methods can be employed. In vitro methods: These include simulated gastrointestinal digestion, artificial membranes, Caco‐2 cell cultures, and isolated or reconstituted cell membranes. Ex vivo methods: These involve lab‐scale gastrointestinal organs. In situ methods: These use animal intestinal perfusion. In vivo methods: These include studies conducted in animals or humans. Each of these approaches provides insights into the bioactivity and functional potential of algae protein in different biological contexts (Carbonell‐Capella et al. 2014).

#### 6.3.1 Digestion

Digestion involves the physical and biochemical breakdown of algae protein, which is essential for its subsequent absorption in the human body. Key organs involved in the degradation of algae protein include the mouth, stomach, and intestine. Initially, the algae food matrix is broken down by chewing in the mouth. This is followed by degradation by pepsin and pepsinogens in the stomach, and by pancreatin in the small intestine (Cian et al. 2015; Wells et al. 2017). To assess the extent of protein digestion, the degree of protein hydrolysis and amino acid bioaccessibility are evaluated (Le Roux 2019). A novel industry‐scale microfluidization technique has been utilized, resulting in a 20% improvement in in vitro digestibility (Ke et al. 2023). Algae protein generally exhibits high digestibility, and novel methods can further enhance this process. For instance, the protein from the microalgae *Spirulina platensis* has been found to be digested at rates as high as 87.5%–97.8% (Ahmad and Ashraf 2023; Yucetepe et al. 2018).

The digestion of algae protein is typically compared to that of animal proteins to assess its potential as an alternative protein source. Algae protein generally has a weaker digestion ability compared to animal protein, such as casein (Wells et al. 2017). Despite this, microalgae‐derived peptides with antihypertensive and antioxidant properties have been produced, which are closely related to peptide extraction methods. For algae protein to become a viable commercial alternative, large‐scale microalgae cultivation, effective peptide release within food products, and evidence of health benefits through digestion are necessary (Ejike et al. 2017). Algae‐derived peptides could offer various health benefits, including antioxidative, antihypertensive, immunomodulatory, anticancer, hepatoprotective, and anticoagulant properties. The potential health benefits of algae peptides have garnered significant research interest. However, most verification studies are still in the laboratory phase, and further research is needed to confirm these benefits on a larger scale (Caporgno and Mathys 2018).

#### 6.3.2 Bioavailability

The bioavailability of proteins is determined by the digestion and absorption of nutrients in the human body. In Becker's (2007) study, the protein efficiency ratio, biological value, net protein utilization, and digestibility of plant proteins were compared to those of animal proteins. Algae protein demonstrated a slightly lower degree of protein digestion compared to casein and egg protein (Becker 2007). In addition, protein bioavailability could be assessed by the protein digestibility corrected amino acid score (PDCAAS) (Jareonsin et al. 2024). The PDCAAS of algae is generally lower than the reference (Shabaka and Moawad 2021). Comparatively, the animal protein like milk, egg, beef, and fish had a higher PDCAAS values (0.92–1.00) than algae protein, which had a wide range of PDCAAS (0.29–0.84) (El Obeid et al. 2025). As for the algae species of low PDCAAS, it is suggested to combine these algae with other plant‐based or animal‐based protein to improve the overall protein digestion in food. In addition, some techniques could be developed to break down the cell wall or protein structure of algae. The bioavailability of algae protein also depends on cultivation conditions. Under mixotrophic conditions, the protein bioaccessibility of *G. sulphuraria* (SAG 108.79) and *G. sulphuraria* (ACUF 064) was 55.3% ± 1.8% and 16.0% ± 1.5%, respectively; in autotrophic conditions, it was 69.3% ± 2.8% and 12.1% ± 1.0%, respectively (Canelli et al. 2023).

#### 6.3.3 Bioactivity

The bioactive compounds in algae are not only proteins; other components such as phenolic compounds also play a significant role (Custódio et al. 2012). Moreover, the bioactivity of algae protein is primarily linked to degraded peptides (Fu et al. 2021; Le Roux 2019). Despite issues related to bitterness, bioavailability, and stability in different environments (Chakrabarti et al. 2018), algae remain a crucial nutritional supplement with numerous health benefits. Extracting protein from biomass can improve its bioaccessibility (Silva et al. 2025). Additionally, bioactive peptides can be recovered from algae protein waste (Sheih et al. 2009). *Spirulina*, for example, has significant applications in peptide products, offering functional activities such as ACE inhibition, antihypertensive effects, DPP‐IV inhibition, and antimicrobial properties (Villaró et al. 2023). To obtain high‐purity bioactive peptides, lipids and colorants are removed before hydrolysis. Peptides smaller than 3 KDa can then be concentrated by filtration, enhancing their bioactivity. Bread, a commonly consumed cereal‐based product, is an ideal matrix for encapsulating bioactive compounds. Similarly, the bioactivity of flour‐based foods is enhanced in some Asian dishes (Prabhasankar et al. 2009). Microalgae from different phyla exhibit antioxidant, metal‐chelating, and acetylcholinesterase‐inhibiting activities (Custódio et al. 2012). Polyphenols, released from disrupted algae cells, enhance antioxidant capabilities, with concentrations increasing alongside algae biomass. Notably, peptides degraded from proteins also possess antioxidant properties (Ejike et al. 2017; Lafarga et al. 2019). For instance, pasta enriched with *Spirulina* exhibits higher antioxidant activity (Rodríguez De Marco et al. 2014). The chelation of Fe^2+^ and Cu^2+^ is influenced by the concentration of algae extracts and species, with a 1 mg/mL concentration achieving a minimum 73% chelating ability. Among various algae species, *N. oculata* demonstrates the best chelating ability (Custódio et al. 2012). Natural peptides from *Tetraselmis suecica* exhibit antibacterial activity without enzymatic effects (Guzmán et al. 2019). Additionally, the antihypertensive activity of algae peptides has proven effective, varying according to algae species and peptide types (Jiang et al. 2021).

## 7 Conclusions and Future Perspectives

Algae represent a sustainable and promising food source due to their production efficiency, functional properties, and nutritional benefits. Unlike traditional agriculture, algae cultivation requires minimal land and has a high capacity for carbon dioxide adsorption, making it an environmentally friendly alternative. Microalgae, in particular, grow rapidly and can survive in extreme environments, making algae farming feasible even in outer space. Consequently, microalgae are promising for interstellar agriculture, serving as a food source for astronauts. The unique functionalities of algae protein form the foundation for its application in food development, offering distinct advantages over plant proteins. Algal protein can be utilized in mixing with animal protein or other plant protein, which has demonstrated unexpectedly superior functionality compared to many isolated proteins. This insight encourages food producers to consider two primary approaches for incorporating algae into food products: using the whole protein biomass or isolated proteins. Ultimately, leveraging the potential of algae protein could enhance the nutritional value of various foods and contribute to more sustainable food production practices.

### 7.1 Cultivation and Production

The cultivation of algae for food purposes is advancing, requiring stringent hygienic conditions. Waste products from algae food processing can be repurposed for biofertilizers, biofuels, or bioplastics. Algae cultivation can be scaled up using unexploited areas such as deserts.

### 7.2 Protein Extraction

Extreme chemical treatments may disrupt the original structure of algae proteins. However, mechanical methods should also be considered to enhance protein extraction. Clarifying the origins and methods of algae protein extraction is crucial, as understanding the relationship between extraction techniques and protein structure is necessary for optimizing the functionality of algae protein. High‐protein biomass or isolated protein products are essential for large‐scale application in the food industry. Future protein extraction efforts should focus on preserving protein functionality rather than solely on extraction efficiency.

### 7.3 Protein Functionality

Enhancing protein functionality can expand the applications of algae. Solubility and emulsification are key functionalities, often prioritized over others. The functional properties of protein isolates differ from those of whole biomass proteins. Isolated algae proteins offer stable functionalities, whereas biomass proteins provide a broader range of functional benefits. The functionality of algae protein is heavily influenced by extraction methods and environmental conditions.

### 7.4 Algae‐Based Food

The development of algae‐based foods is in its early stages, with algae protein primarily used in meat substitutes. Increasing research is being conducted on aquaculture feed and pet food, where techniques from plant‐based foods can be adapted. However, developing algae foods involves addressing complex issues of color, flavor, and taste. Currently, filtration and isolation of algae proteins appear to be feasible solutions. Beverages fortified with hydrophilic amino acids demonstrate enhanced nutritional value.

### 7.5 Food Safety

Food authorities in various countries ensure the safety of algae as a food source. Special attention must be given to the cultivation environment, particularly when algae are grown in open spaces like ponds. If chemical extraction methods are used, it is crucial to manage salt intake or employ filtration techniques to reduce salt levels in the final product.

## Author Contributions

Shaozong Wu wrote the manuscript. Shaozong Wu and Christelle Turchiuli collected and reviewed the literature while acting on the acquisition of funding. Christelle Turchiuli, Paul Menut, and Song Miao contributed to the analysis of the documents used and to the revision of the manuscript. All co‐authors read and approved the final manuscript.

## Conflicts of Interest

The authors declare no conflicts of interest.

## References

[crf370264-bib-0001] Abiusi, F. , P. Moñino Fernández , S. Canziani , M. Janssen , R. H. Wijffels , and M. Barbosa . 2022. “Mixotrophic Cultivation of *Galdieria sulphuraria* for C‐Phycocyanin and Protein Production.” Algal Research 61: 102603. 10.1016/j.algal.2021.102603.

[crf370264-bib-0002] Ahmad, A. , and S. S. Ashraf . 2023. “Sustainable Food and Feed Sources From Microalgae: Food Security and the Circular Bioeconomy.” Algal Research 74: 103185. 10.1016/j.algal.2023.103185.

[crf370264-bib-0003] Alekseeva, E. , and V. Kolchina . 2019. “Amino Acid Composition of Beef Obtained Fromthe Specialized Meat Cattle.” IOP Conference Series: Earth and Environmental Science 341, no. 1: 012136. 10.1088/1755-1315/341/1/012136.

[crf370264-bib-0004] Amagliani, L. , and C. Schmitt . 2017. “Globular Plant Protein Aggregates for Stabilization of Food Foams and Emulsions.” Trends in Food Science & Technology 67: 248–259. 10.1016/j.tifs.2017.07.013.

[crf370264-bib-0005] Amagliani, L. , J. V. C. Silva , M. Saffon , and J. Dombrowski . 2021. “On the Foaming Properties of Plant Proteins: Current Status and Future Opportunities.” Trends in Food Science & Technology 118: 261–272. 10.1016/j.tifs.2021.10.001.

[crf370264-bib-0006] Araújo, R. , F. Vázquez Calderón , J. Sánchez López , et al. 2021. “Current Status of the Algae Production Industry in Europe: an Emerging Sector of the Blue Bioeconomy.” Frontiers in Marine Science 7: 626389. 10.3389/fmars.2020.626389.

[crf370264-bib-0007] Attia, Y. A. , M. A. Al‐Harthi , M. A. Korish , and M. H. Shiboob . 2020. “Protein and Amino Acid Content in Four Brands of Commercial Table Eggs in Retail Markets in Relation to Human Requirements.” Animals (Basel) 10, no. 3: 406. 10.3390/ani10030406. 32121495 PMC7142600

[crf370264-bib-0008] Aykan, N. F. 2015. “Red Meat and Colorectal Cancer.” Oncology Reviews 9, no. 1: 288. 10.4081/oncol.2015.288. 26779313 PMC4698595

[crf370264-bib-0009] Ba, F. , A. V. Ursu , C. Laroche , and G. Djelveh . 2016. “ *Haematococcus pluvialis* Soluble Proteins: Extraction, Characterization, Concentration/Fractionation and Emulsifying Properties.” Bioresource Technology 200: 147–152. 10.1016/j.biortech.2015.10.012. 26476616

[crf370264-bib-0010] Barba, F. J. 2017. “Microalgae and Seaweeds for Food Applications: Challenges and Perspectives.” Food Research International 99, no. Pt 3: 969–970. 10.1016/j.foodres.2016.12.022. 28865622

[crf370264-bib-0011] Becker, E. W. 2007. “Micro‐Algae as a Source of Protein.” Biotechnology Advances 25, no. 2: 207–210. 10.1016/j.biotechadv.2006.11.002. 17196357

[crf370264-bib-0012] Beheshtipour, H. , A. M. Mortazavian , R. Mohammadi , S. Sohrabvandi , and K. Khosravi‐Darani . 2013. “Supplementation of *Spirulina platensis* and *Chlorella vulgaris* Algae Into Probiotic Fermented Milks.” Comprehensive Reviews in Food Science and Food Safety 12, no. 2: 144–154. 10.1111/1541-4337.12004.

[crf370264-bib-0013] Benelhadj, S. , A. Gharsallaoui , P. Degraeve , H. Attia , and D. Ghorbel . 2016. “Effect of pH on the Functional Properties of *Arthrospira* (Spirulina) *platensis* Protein Isolate.” Food Chemistry 194: 1056–1063. 10.1016/j.foodchem.2015.08.133. 26471653

[crf370264-bib-0014] Bernaerts, T. M. M. , A. Panozzo , V. Doumen , et al. 2017. “Microalgal Biomass as a (multi)Functional Ingredient in Food Products: Rheological Properties of Microalgal Suspensions as Affected by Mechanical and Thermal Processing.” Algal Research 25: 452–463. 10.1016/j.algal.2017.05.014.

[crf370264-bib-0015] Bertsch, P. , L. Böcker , A.‐S. Palm , J. Bergfreund , P. Fischer , and A. Mathys . 2023. “ *Arthrospira platensis* Protein Isolate for Stabilization of Fluid Interfaces: Effect of Physicochemical Conditions and Comparison to Animal‐Based Proteins.” Food Hydrocolloids 136: 108290. 10.1016/j.foodhyd.2022.108290.

[crf370264-bib-0016] Bhatnagar, P. , P. Gururani , N. Singh , P. Gautam , M. S. Vlaskin , and V. Kumar . 2023. “Review on Microalgae Protein and Its Current and Future Utilisation in the Food Industry.” International Journal of Food Science & Technology 59: 473–480. 10.1111/ijfs.16586.

[crf370264-bib-0017] Bjarnadóttir, M. , B. V. Aðalbjörnsson , A. Nilsson , et al. 2018. “ *Palmaria palmata* as an Alternative Protein Source: Enzymatic Protein Extraction, Amino Acid Composition, and Nitrogen‐to‐Protein Conversion Factor.” Journal of Applied Phycology 30, no. 3: 2061–2070. 10.1007/s10811-017-1351-8.

[crf370264-bib-0018] Bleakley, S. , and M. Hayes . 2017. “Algal Proteins: Extraction, Application, and Challenges Concerning Production.” Foods 6, no. 5: 33. 10.3390/foods6050033. 28445408 PMC5447909

[crf370264-bib-0019] Böcker, L. , P. Bertsch , D. Wenner , et al. 2021. “Effect of *Arthrospira platensis* Microalgae Protein Purification on Emulsification Mechanism and Efficiency.” Journal of Colloid & Interface Science 584: 344–353. 10.1016/j.jcis.2020.09.067. 33070074

[crf370264-bib-0020] Bosnea, L. , A. Terpou , E. Pappa , et al. 2021. “Incorporation of *Spirulina platensis* on Traditional Greek Soft Cheese With Respect to Its Nutritional and Sensory Perspectives.” Proceedings 70, no. 1: 99. 10.3390/foods_2020-07600.

[crf370264-bib-0021] Boukid, F. , and M. Gagaoua . 2022. “Vegan Egg: A Future‐Proof Food Ingredient?” Foods 11, no. 2: 161. 10.3390/foods11020161. 35053893 PMC8774821

[crf370264-bib-0022] Boukid, F. , C. M. Rosell , S. Rosene , S. Bover‐Cid , and M. Castellari . 2021. “Non‐Animal Proteins as Cutting‐Edge Ingredients to Reformulate Animal‐Free Foodstuffs: Present Status and Future Perspectives.” Critical Reviews in Food Science and Nutrition 62: 6390–6420. 10.1080/10408398.2021.1901649. 33775185

[crf370264-bib-0023] Buchmann, L. , P. Bertsch , L. Böcker , U. Krähenmann , P. Fischer , and A. Mathys . 2019. “Adsorption Kinetics and Foaming Properties of Soluble Microalgae Fractions at the Air/Water Interface.” Food Hydrocolloids 97: 105182. 10.1016/j.foodhyd.2019.105182.

[crf370264-bib-0024] Canelli, G. , F. Abiusi , A. Vidal Garcia , S. Canziani , and A. Mathys . 2023. “Amino Acid Profile and Protein Bioaccessibility of Two *Galdieria sulphuraria* Strains Cultivated Autotrophically and Mixotrophically in Pilot‐Scale Photobioreactors.” Innovative Food Science & Emerging Technologies 84: 103287. 10.1016/j.ifset.2023.103287.

[crf370264-bib-0025] Caporgno, M. P. , L. Böcker , C. Müssner , et al. 2020. “Extruded Meat Analogues Based on Yellow, Heterotrophically Cultivated *Auxenochlorella protothecoides* Microalgae.” Innovative Food Science & Emerging Technologies 59: 102275. 10.1016/j.ifset.2019.102275.

[crf370264-bib-0026] Caporgno, M. P. , and A. Mathys . 2018. “Trends in Microalgae Incorporation Into Innovative Food Products With Potential Health Benefits.” Frontiers in Nutrition 5: 58. 10.3389/fnut.2018.00058. 30109233 PMC6080594

[crf370264-bib-0027] Carbonell‐Capella, J. M. , M. Buniowska , F. J. Barba , M. J. Esteve , and A. Frígola . 2014. “Analytical Methods for Determining Bioavailability and Bioaccessibility of Bioactive Compounds From Fruits and Vegetables: A Review.” Comprehensive Reviews in Food Science and Food Safety 13, no. 2: 155–171. 10.1111/1541-4337.12049. 33412647

[crf370264-bib-0028] Carullo, D. , B. D. Abera , A. A. Casazza , et al. 2018. “Effect of Pulsed Electric Fields and High Pressure Homogenization on the Aqueous Extraction of Intracellular Compounds From the Microalgae *Chlorella vulgaris* .” Algal Research 31: 60–69. 10.1016/j.algal.2018.01.017.

[crf370264-bib-0029] Carullo, D. , F. Donsì , G. Ferrari , and G. Pataro . 2021. “Extraction Improvement of Water‐Soluble Compounds From *Arthrospira platensis* Through the Combination of High‐Shear Homogenization and Pulsed Electric Fields.” Algal Research 57: 102341. 10.1016/j.algal.2021.102341.

[crf370264-bib-0030] Cavonius, L. R. , E. Albers , and I. Undeland . 2015. “pH‐Shift Processing of *Nannochloropsis oculata* Microalgal Biomass to Obtain a Protein‐Enriched Food or Feed Ingredient.” Algal Research 11: 95–102. 10.1016/j.algal.2015.05.022.

[crf370264-bib-0031] Chakrabarti, S. , S. Guha , and K. Majumder . 2018. “Food‐Derived Bioactive Peptides in Human Health: Challenges and Opportunities.” Nutrients 10, no. 11: 1738. 10.3390/nu10111738. 30424533 PMC6265732

[crf370264-bib-0032] Chen, Y. , J. Chen , C. Chang , et al. 2019. “Physicochemical and Functional Properties of Proteins Extracted From Three Microalgal Species.” Food Hydrocolloids 96: 510–517. 10.1016/j.foodhyd.2019.05.025.

[crf370264-bib-0033] Chronakis, I. S. 2001. “Gelation of Eedible Blue‐Green Algae Protein Isolate (*Spirulina platensis* Strain Pacifica): Thermal Transitions, Rheological Properties, and Molecular Forces Involved.” Journal of Agriculture and Food Chemistry 49: 888–898. 10.1021/jf0005059 11262046

[crf370264-bib-0034] Cian, R. , S. Drago , F. De Medina , and O. Martínez‐Augustin . 2015. “Proteins and Carbohydrates From Red Seaweeds: Evidence for Beneficial Effects on Gut Function and Microbiota.” Marine Drugs 13, no. 8: 5358–5383. 26308006 10.3390/md13085358 PMC4557026

[crf370264-bib-0035] Custódio, L. , T. Justo , L. Silvestre , et al. 2012. “Microalgae of Different Phyla Display Antioxidant, Metal Chelating and Acetylcholinesterase Inhibitory Activities.” Food Chemistry 131, no. 1: 134–140. 10.1016/j.foodchem.2011.08.047.

[crf370264-bib-0036] Dabbour, M. , A. Hamoda , H. Xu , et al. 2024. “pH‐Shifting and Sonication Synergistically Altered Cottonseed Protein: Correlating the Conformational and Functional Characteristics.” Industrial Crops and Products 222: 120043. 10.1016/j.indcrop.2024.120043.

[crf370264-bib-0038] Dai, L. , J. Hinrichs , and J. Weiss . 2020a. “Ionic Strength and pH Stability of Oil‐in‐Water Emulsions Prepared With Acid‐Hydrolyzed Insoluble Proteins From *Chlorella protothecoides* .” Journal of the Science of Food and Agriculture 100, no. 11: 4237–4244. 10.1002/jsfa.10464. 32378211

[crf370264-bib-0039] Dai, L. , J. Hinrichs , and J. Weiss . 2020b. “Emulsifying Properties of Acid‐Hydrolyzed Insoluble Protein Fraction From *Chlorella protothecoides*: Formation and Storage Stability of Emulsions.” Food Hydrocolloids 108, no. 2: 105954. 10.1016/j.foodhyd.2020.105954.

[crf370264-bib-0040] Dai, L. , R. Shivananda , J. Hinrichs , and J. Weiss . 2020. “Foaming of Acid‐Hydrolyzed Insoluble Microalgae Proteins From *Chlorella protothecoides* .” Food Biophysics 15, no. 3: 368–375. 10.1007/s11483-020-09630-2.

[crf370264-bib-0040a] Dai, L. , M. Cepeda , J. Hinrichs , and J. Weiss . 2021. “Behavior of Concentrated Emulsions Prepared by Acid‐Hydrolyzed Insoluble Microalgae Proteins From *Chlorella protothecoides* .” Journal of the Science of Food and Agriculture 101, no. 8: 3348–3354. 10.1002/jsfa.10964. 33222184

[crf370264-bib-0041] Dalle Zotte, A. , R. Ricci , M. Cullere , L. Serva , S. Tenti , and G. Marchesini . 2020. “Research Note: Effect of Chicken Genotype and White Striping‐Wooden Breast Condition on Breast Meat Proximate Composition and Amino Acid Profile.” Poultry Science 99, no. 3: 1797–1803. 10.1016/j.psj.2019.10.066. PMC7587648 32115042

[crf370264-bib-0042] Deepika, C. , J. Wolf , J. Roles , I. Ross , and B. Hankamer . 2022. “Sustainable Production of Pigments From Cyanobacteria.” In Cyanobacteria in Biotechnology, 171–251. Springer. 10.1007/10_2022_211 36571616

[crf370264-bib-0043] de Farias Neves, F. , M. Demarco , and G. Tribuzi . 2019. “Drying and Quality of Microalgal Powders for Human Alimentation.” In Microalgae‐From Physiology to Application. IntechOpen, 1‐20.

[crf370264-bib-0044] de Medeiros, V. P. B. , T. C. Pimentel , A. S. Sant'Ana , and M. Magnani . 2021. “Microalgae in the Meat Processing Chain: Feed for Animal Production or Source of Techno‐Functional Ingredients.” Current Opinion in Food Science 37: 125–134. 10.1016/j.cofs.2020.10.014.

[crf370264-bib-0045] Ebert, S. , L. Grossmann , J. Hinrichs , and J. Weiss . 2019. “Emulsifying Properties of Water‐Soluble Proteins Extracted From the Microalgae *Chlorella sorokiniana* and *Phaeodactylum tricornutum* .” Food & Function 10, no. 2: 754–764. 30667441 10.1039/c8fo02197j

[crf370264-bib-0046] Ejike, C. E. C. C. , S. A. Collins , N. Balasuriya , A. K. Swanson , B. Mason , and C. C. Udenigwe . 2017. “Prospects of Microalgae Proteins in Producing Peptide‐Based Functional Foods for Promoting Cardiovascular Health.” Trends in Food Science & Technology 59: 30–36. 10.1016/j.tifs.2016.10.026.

[crf370264-bib-0047] El Obeid, T. , İ. Atalar , O. S. Toker , I. Palabiyik , and Y. F. Gorgulu . 2025. “Modification and Glycation Microalgae Proteins by Non‐Thermal Assisted Process.” Current Opinion in Food Science 62: 101263. 10.1016/j.cofs.2024.101263.

[crf370264-bib-0048] Erem, E. , and M. Kilic‐Akyilmaz . 2024. “The Role of Fermentation With Lactic Acid Bacteria in Quality and Health Effects of Plant‐Based Dairy Analogues.” Comprehensive Reviews in Food Science and Food Safety 23, no. 4: e13402. 10.1111/1541-4337.13402. 39030804

[crf370264-bib-0049] Espinosa‐Ramírez, J. , A. C. Mondragón‐Portocarrero , J. A. Rodríguez , J. M. Lorenzo , and E. M. Santos . 2023. “Algae as a Potential Source of Protein Meat Alternatives.” Frontiers in Nutrition 10: 1254300. 10.3389/fnut.2023.1254300. 37743912 PMC10513374

[crf370264-bib-0050] European Commission . 2018. A Sustainable Bioeconomy for Europe: Strengthening the Connection Between Economy, Society and the Environment . European Commission. 10.2777/792130.

[crf370264-bib-0051] European Parliament . 2023. The Future of the EU Algae Sector, Policy Department for Structural and Cohesion Policies Directorate‐General for Internal Policies. European Parliament.

[crf370264-bib-0052] Feng, G. X. , G. S. Wang , Q. Li , et al. 2023. “Depletion Attraction Driven Formation of Spirulina Emulsion Gels for 3D Printing.” Food Hydrocolloids 141: 108691. 10.1016/j.foodhyd.2023.108691.

[crf370264-bib-0053] Friedman, M. 1996. “Nutritional Value of Proteins From Different Food Sources. A Review.” Journal of Agricultural and Food Chemistry 44, no. 1: 6–29. 10.1021/jf9400167.

[crf370264-bib-0054] Fu, Y. , T. Chen , S. H. Y. Chen , et al. 2021. “The Potentials and Challenges of Using Microalgae as an Ingredient to Produce Meat Analogues.” Trends in Food Science & Technology 112: 188–200. 10.1016/j.tifs.2021.03.050.

[crf370264-bib-0055] Garcia‐Vaquero, M. , M. Lopez‐Alonso , and M. Hayes . 2017. “Assessment of the Functional Properties of Protein Extracted From the Brown Seaweed *Himanthalia elongata* (Linnaeus) S. F. Gray.” Food Research International 99, no. Pt 3: 971–978. 10.1016/j.foodres.2016.06.023. 28865623

[crf370264-bib-0056] Geada, P. , C. Moreira , M. Silva , et al. 2021. “Algal Proteins: Production Strategies and Nutritional and Functional Properties.” Bioresource Technology 332: 125125. 10.1016/j.biortech.2021.125125. 33865652

[crf370264-bib-0057] Gerde, J. A. , T. Wang , L. Yao , S. Jung , L. A. Johnson , and B. Lamsal . 2013. “Optimizing Protein Isolation From Defatted and Non‐Defatted Nannochloropsis Microalgae Biomass.” Algal Research 2, no. 2: 145–153. 10.1016/j.algal.2013.02.001.

[crf370264-bib-0058] Gorissen, S. H. M. , J. J. R. Crombag , J. M. G. Senden , et al. 2018. “Protein Content and Amino Acid Composition of Commercially Available Plant‐Based Protein Isolates.” Amino Acids 50, no. 12: 1685–1695. 10.1007/s00726-018-2640-5. 30167963 PMC6245118

[crf370264-bib-0059] Górska‐Warsewicz, H. , K. Rejman , W. Laskowski , and M. Czeczotko . 2019. “Milk and Dairy Products and Their Nutritional Contribution to the Average Polish Diet.” Nutrients 11, no. 8: 1771. 10.3390/nu11081771. 31374893 PMC6723869

[crf370264-bib-0060] Grahame, A. 2023. *European Market Insights, China's Food Policy, State of the Market Report and More*. Plant Based World Pulse, April 24, 2023. https://plantbasedworldpulse.com/european‐market‐insights‐chinas‐food‐policy‐state‐of‐the‐market‐report‐and‐more/.

[crf370264-bib-0061] Grahl, S. , M. Palanisamy , M. Strack , L. Meier‐Dinkel , S. Toepfl , and D. Mörlein . 2018. “Towards More Sustainable Meat Alternatives: How Technical Parameters Affect the Sensory Properties of Extrusion Products Derived From Soy and Algae.” Journal of Cleaner Production 198: 962–971. 10.1016/j.jclepro.2018.07.041.

[crf370264-bib-0062] Grossmann, L. , S. Ebert , J. Hinrichs , and J. Weiss . 2018. “Production of Protein‐Rich Extracts From Disrupted Microalgae Cells: Impact of Solvent Treatment and Lyophilization.” Algal Research 36: 67–76. 10.1016/j.algal.2018.09.011.

[crf370264-bib-0063] Grossmann, L. , J. Hinrichs , H. D. Goff , and J. Weiss . 2019. “Heat‐Induced Gel Formation of a Protein‐Rich Extract From the Microalga *Chlorella sorokiniana* .” Innovative Food Science & Emerging Technologies 56: 102176. 10.1016/j.ifset.2019.06.001.

[crf370264-bib-0064] Grossmann, L. , J. Hinrichs , and J. Weiss . 2019. “Solubility and Aggregation Behavior of Protein Fractions From the Heterotrophically Cultivated Microalga *Chlorella protothecoides* .” Food Research International 116: 283–290. 10.1016/j.foodres.2018.08.037. 30716947

[crf370264-bib-0065] Grossmann, L. , and D. J. McClements . 2022. “Current Insights Into Protein Solubility: A Review of Its Importance for Alternative Proteins.” Food Hydrocolloids 137: 108416. 10.1016/j.foodhyd.2022.108416.

[crf370264-bib-0066] Guzmán, F. , G. Wong , T. Román , et al. 2019. “Identification of Antimicrobial Peptides From the Microalgae *Tetraselmis suecica* (Kylin) Butcher and Bactericidal Activity Improvement.” Marine Drugs 17, no. 8: 453. 10.3390/md17080453. 31374937 PMC6722934

[crf370264-bib-0067] Hernández, H. , M. C. Nunes , C. Prista , and A. Raymundo . 2022. “Innovative and Healthier Dairy Products Through the Addition of Microalgae: A Review.” Foods 11, no. 5: 755. 10.3390/foods11050755. 35267388 PMC8909392

[crf370264-bib-0068] Hildebrand, G. , M. M. Poojary , C. O'Donnell , M. N. Lund , M. Garcia‐Vaquero , and B. K. Tiwari . 2020. “Ultrasound‐Assisted Processing of *Chlorella vulgaris* for Enhanced Protein Extraction.” Journal of Applied Phycology 32, no. 3: 1709–1718. 10.1007/s10811-020-02105-4.

[crf370264-bib-0069] Jareonsin, S. , C. Pumas , D. Jaitiang , and T. Uttarotai . 2024. “Green Fusion Proteins: an Approach to Sustainable Nutrition Blending Plant and Algae‐Based Proteins for a Circular Food System.” Future Foods 10: 100415. 10.1016/j.fufo.2024.100415.

[crf370264-bib-0070] Jayaprakash, P. , A. Maudhuit , C. Gaiani , and S. Desobry . 2023. “Encapsulation of Bioactive Compounds Using Competitive Emerging Techniques: Electrospraying, Nano Spray Drying, and Electrostatic Spray Drying.” Journal of Food Engineering 339: 111260. 10.1016/j.jfoodeng.2022.111260.

[crf370264-bib-0071] Jiang, Q. , Q. Chen , T. Zhang , M. Liu , S. Duan , and X. Sun . 2021. “The Antihypertensive Effects and Potential Molecular Mechanism of Microalgal Angiotensin I‐Converting Enzyme Inhibitor‐Like Peptides: A Mini Review.” International Journal of Molecular Sciences 22, no. 8: 4068. 10.3390/ijms22084068. 33920763 PMC8071128

[crf370264-bib-0072] Kadam, S. U. , C. Álvarez , B. K. Tiwari , and C. P. O'Donnell . 2017. “Extraction and Characterization of Protein From Irish Brown Seaweed *Ascophyllum nodosum* .” Food Research International 99, no. Pt 3: 1021–1027. 10.1016/j.foodres.2016.07.018. 28865612

[crf370264-bib-0073] Ke, Y. , J. Chen , T. Dai , et al. 2023. “Developing Industry‐Scale Microfluidization for Cell Disruption, Biomolecules Release and Bioaccessibility Improvement of Chlorella Pyrenoidosa.” Bioresource Technology 387: 129649. 10.1016/j.biortech.2023.129649. 37558104

[crf370264-bib-0074] Khaled, B. M. , A. K. Das , S. M. S. Alam , et al. 2024. “Effect of Different Drying Techniques on the Physicochemical and Nutritional Properties of *Moringa oleifera* Leaves Powder and Their Application in Bakery Product.” Applied Food Research 4, no. 2: 100599. 10.1016/j.afres.2024.100599.

[crf370264-bib-0075] Köhler, R. , L. Kariuki , C. Lambert , and H. K. Biesalski . 2019. “Protein, Amino Acid and Mineral Composition of Some Edible Insects From Thailand.” Journal of Asia‐Pacific Entomology 22, no. 1: 372–378. 10.1016/j.aspen.2019.02.002.

[crf370264-bib-0076] Lafarga, T. , F. G. Acién‐Fernández , M. Castellari , S. Villaró , G. Bobo , and I. Aguiló‐Aguayo . 2019. “Effect of Microalgae Incorporation on the Physicochemical, Nutritional, and Sensorial Properties of an Innovative Broccoli Soup.” Lwt 111: 167–174. 10.1016/j.lwt.2019.05.037.

[crf370264-bib-0077] Lam, G. P. 'T. , P. R. Postma , D. A. Fernandes , et al. 2017. “Pulsed Electric Field for Protein Release of the Microalgae *Chlorella vulgaris* and *Neochloris oleoabundans* .” Algal Research 24: 181–187. 10.1016/j.algal.2017.03.024.

[crf370264-bib-0078] Landi, N. , S. Ragucci , and A. Di Maro . 2021. “Amino Acid Composition of Milk From Cow, Sheep and Goat Raised in Ailano and Valle Agricola, Two Localities of ‘Alto Casertano’ (Campania Region).” Foods 10, no. 10: 2431. 10.3390/foods10102431. 34681478 PMC8535404

[crf370264-bib-0079] Le Roux, L. 2019. De la fabrication à la digestion in vitro de formules infantiles innovantes en partie composées de protéines végétales: Une approche multi‐échelle. Agrocampus Ouest , COMUE Université Bretagne Loire.

[crf370264-bib-0080] Le Roux, L. , S. Mejean , R. Chacon , et al. 2020. “Plant Proteins Partially Replacing Dairy Proteins Greatly Influence Infant Formula Functionalities.” Lwt 120: 108891. 10.1016/j.lwt.2019.108891.

[crf370264-bib-0081] Li, R. , T. K. B. Mejdahl , M. L. Jørgensen , M. Corredig , and S. B. Gregersen . 2024. “Effect of Processing on Protein Solubility of *Chlorella sorokiniana* Dispersions.” Sustainable Food Proteins 2, no. 4: 215–222. 10.1002/sfp2.1037.

[crf370264-bib-0082] Li, S. , M. Luo , D. Wannasin , et al. 2024. “Exploring the Potential of Plant‐Based Emulsion Gels Enriched With β‐Glucan and Potato Protein as Egg Yolk Alternatives.” Food Hydrocolloids 148: 109511. 10.1016/j.foodhyd.2023.109511.

[crf370264-bib-0083] Liu, S. , I. Gifuni , H. Mear , M. Frappart , and E. Couallier . 2021. “Recovery of Soluble Proteins From *Chlorella vulgaris* by Bead‐Milling and Microfiltration: Impact of the Concentration and the Physicochemical Conditions During the Cell Disruption on the Whole Process.” Process Biochemistry 108: 34–47. 10.1016/j.procbio.2021.05.021.

[crf370264-bib-0084] Liu, X. , J. Wang , Q. Huang , et al. 2020. “Underlying Mechanism for the Differences in Heat‐Induced Gel Properties Between Thick Egg Whites and Thin Egg Whites: Gel Properties, Structure and Quantitative Proteome Analysis.” Food Hydrocolloids 106: 105873. 10.1016/j.foodhyd.2020.105873.

[crf370264-bib-0085] Lorente, E. , M. Hapońska , E. Clavero , C. Torras , and J. Salvadó . 2017. “Microalgae Fractionation Using Steam Explosion, Dynamic and Tangential Cross‐Flow Membrane Filtration.” Bioresource Technology 237: 3–10. 10.1016/j.biortech.2017.03.129. 28395932

[crf370264-bib-0086] Malla, N. , J. V. Nørgaard , H. N. Lærke , L.‐H. L. Heckmann , and N. Roos . 2022. “Some Insect Species Are Good‐Quality Protein Sources for Children and Adults: Digestible Indispensable Amino Acid Score (DIAAS) Determined in Growing Pigs.” Journal of Nutrition 152, no. 4: 1042–1051. 10.1093/jn/nxac019. 35102372

[crf370264-bib-0087] Marti‐Quijal, F. J. , S. Zamuz , F. Galvez , et al. 2018. “Replacement of Soy Protein With Other Legumes or Algae in Turkey Breast Formulation: Changes in Physicochemical and Technological Properties.” Journal of Food Processing and Preservation 42, no. 12: e13845. 10.1111/jfpp.13845.

[crf370264-bib-0088] Marti‐Quijal, F. J. , S. Zamuz , I. Tomašević , et al. 2019. “Influence of Different Sources of Vegetable, Whey and Microalgae Proteins on the Physicochemical Properties and Amino Acid Profile of Fresh Pork Sausages.” Lwt 110: 316–323. 10.1016/j.lwt.2019.04.097.

[crf370264-bib-0089] Matos, J. , C. Afonso , C. Cardoso , M. L. Serralheiro , and N. M. Bandarra . 2021. “Yogurt Enriched With Isochrysis Galbana: An Innovative Functional Food.” Foods 10, no. 7: 1458. 10.3390/foods10071458. 34202539 PMC8306745

[crf370264-bib-0090] McClements, D. J. 2020. “Development of Next‐Generation Nutritionally Fortified Plant‐Based Milk Substitutes: Structural Design Principles.” Foods 9, no. 4: 421. 10.3390/foods9040421. 32260061 PMC7231295

[crf370264-bib-0091] McClements, D. J. 2023. “Modeling the Rheological Properties of Plant‐Based Foods: Soft Matter Physics Principles.” Sustainable Food Proteins 1: 101–132. 10.1002/sfp2.1015.

[crf370264-bib-0092] McClements, D. J. , and L. Grossmann . 2023. “Next‐Generation Plant‐Based Foods: Challenges and Opportunities.” Annual Review of Food Science and Technology 15, no. 1: 79–101. 10.1146/annurev-food-072023-034414 37963430

[crf370264-bib-0093] Mendes, M. , S. Navalho , A. Ferreira , et al. 2022. “Algae as Food in Europe: An Overview of Species Diversity and Their Application.” Foods 11, no. 13: 1871. 10.3390/foods11131871. 35804686 PMC9265617

[crf370264-bib-0094] Mishra, K. , J. Bergfreund , P. Bertsch , P. Fischer , and E. J. Windhab . 2020. “Crystallization‐Induced Network Formation of Tri‐and Monopalmitin at the Middle‐Chain Triglyceride Oil/Air Interface.” Langmuir: The ACS Journal of Surfaces and Colloids 36, no. 26: 7566–7572. 32520568 10.1021/acs.langmuir.0c01195

[crf370264-bib-0095] Mišurcová, L. , F. Buňka , J. Vávra Ambrožová , L. Machů , D. Samek , and S. Kráčmar . 2014. “Amino Acid Composition of Algal Products and Its Contribution to RDI.” Food Chemistry 151: 120–125. 10.1016/j.foodchem.2013.11.040. 24423510

[crf370264-bib-0096] Mohammed, H. O. , M. N. O'Grady , M. G. O'Sullivan , R. M. Hamill , K. N. Kilcawley , and J. P. Kerry . 2021. “An Assessment of Selected Nutritional, Bioactive, Thermal and Technological Properties of Brown and Red Irish Seaweed Species.” Foods 10, no. 11: 2784. 10.3390/foods10112784. 34829067 PMC8625894

[crf370264-bib-0097] Montalescot, V. , T. Rinaldi , R. Touchard , et al. 2015. “Optimization of Bead Milling Parameters for the Cell Disruption of Microalgae: Process Modeling and Application to *Porphyridium cruentum* and *Nannochloropsis oculata* .” Bioresource Technology 196: 339–346. 10.1016/j.biortech.2015.07.075. 26253918

[crf370264-bib-0098] Muñoz‐Tebar, N. , L. Ong , C. J. Gamlath , et al. 2022. “Nutrient Enrichment of Dairy Curd by Incorporation of Whole and Ruptured Microalgal Cells (*Nannochloropsis salina*).” Innovative Food Science & Emerging Technologies 82: 103211. 10.1016/j.ifset.2022.103211.

[crf370264-bib-0099] Murray, B. S. 2020. “Recent Developments in Food Foams.” Current Opinion in Colloid & Interface Science 50: 101394. 10.1016/j.cocis.2020.101394.

[crf370264-bib-0100] Nie, J. , X. Fu , L. Wang , J. Xu , and X. Gao . 2023. “Impact of *Monascus purpureus* Fermentation on Antioxidant Activity, Free Amino Acid Profiles and Flavor Properties of Kelp (*Saccharina japonica*).” Food Chemistry 400: 133990. 10.1016/j.foodchem.2022.133990. 36063678

[crf370264-bib-0101] Noreen, A. , S. Mahmood , I. Aziz , et al. 2021. “Microalgae as Potential Protein Sources: Evidence From Protein Extraction and Amino Acid Profiling of *Chlorella vulgaris* and *Scenedesmus* sp.” Pakistan Journal of Agricultural Sciences 58, no. 3: 821–829. 10.21162/Pakjas/21.511.

[crf370264-bib-0102] O' Connor, J. , S. Meaney , G. A. Williams , and M. Hayes . 2020. “Extraction of Protein From Four Different Seaweeds Using Three Different Physical Pre‐Treatment Strategies.” Molecules (Basel, Switzerland) 25, no. 8: 2005. 10.3390/molecules25082005. 32344706 PMC7221823

[crf370264-bib-0103] O'Sullivan, A. M. , Y. C. O'Callaghan , M. N. O'Grady , et al. 2014. “An Examination of the Potential of Seaweed Extracts as Functional Ingredients in Milk.” International Journal of Dairy Technology 67, no. 2: 182–193. 10.1111/1471-0307.12121.

[crf370264-bib-0104] Packer, M. A. , G. C. Harris , and S. L. Adams . 2016. “Food and Feed Applications of Algae.” In Algae Biotechnology, 217–247. Springer.

[crf370264-bib-0105] Parniakov, O. , S. Toepfl , F. J. Barba , et al. 2018. “Impact of the Soy Protein Replacement by Legumes and Algae Based Proteins on the Quality of Chicken Rotti.” Journal of Food Science and Technology 55, no. 7: 2552–2559. 10.1007/s13197-018-3175-1. 30042571 PMC6033791

[crf370264-bib-0106] Pereira, A. M. , C. R. Lisboa , and J. A. V. Costa . 2018. “High Protein Ingredients of Microalgal Origin: Obtainment and Functional Properties.” Innovative Food Science & Emerging Technologies 47: 187–194. 10.1016/j.ifset.2018.02.015.

[crf370264-bib-0107] Pereira, H. , J. Silva , T. Santos , et al. 2019. “Nutritional Potential and Toxicological Evaluation of *Tetraselmis* sp. CTP4 Microalgal Biomass Produced in Industrial Photobioreactors.” Molecules (Basel, Switzerland) 24, no. 17: 3192. 10.3390/molecules24173192. 31484299 PMC6749414

[crf370264-bib-0108] Prabhasankar, P. , P. Ganesan , N. Bhaskar , et al. 2009. “Edible Japanese Seaweed, Wakame (*Undaria pinnatifida*) as an Ingredient in Pasta: Chemical, Functional and Structural Evaluation.” Food Chemistry 115, no. 2: 501–508. 10.1016/j.foodchem.2008.12.047.

[crf370264-bib-0109] Rachwa‐Rosiak, D. , E. Nebesny , and G. Budryn . 2015. “Chickpeas‐Composition, Nutritional Value, Health Benefits, Application to Bread and Snacks: A Review.” Critical Reviews in Food Science and Nutrition 55, no. 8: 1137–1145. 10.1080/10408398.2012.687418. 24915347

[crf370264-bib-0110] Raczyk, M. , K. Polanowska , B. Kruszewski , A. Grygier , and D. Michałowska . 2022. “Effect of Spirulina (*Arthrospira platensis*) Supplementation on Physical and Chemical Properties of Semolina (*Triticum durum*) Based Fresh Pasta.” Molecules (Basel, Switzerland) 27, no. 2: 355. 10.3390/molecules27020355. 35056669 PMC8779242

[crf370264-bib-0111] Ritchie, H. , P. Rosado , and M. Roser . 2017. Meat and Dairy Production. Our World in Data.

[crf370264-bib-0112] Rodríguez De Marco, E. , M. E. Steffolani , C. S. Martínez , and A. E. León . 2014. “Effects of Spirulina Biomass on the Technological and Nutritional Quality of Bread Wheat Pasta.” LWT—Food Science and Technology 58, no. 1: 102–108. 10.1016/j.lwt.2014.02.054.

[crf370264-bib-0113] Roohinejad, S. , M. Koubaa , F. J. Barba , S. Saljoughian , M. Amid , and R. Greiner . 2017. “Application of Seaweeds to Develop New Food Products With Enhanced Shelf‐Life, Quality and Health‐Related Beneficial Properties.” Food Research International 99, no. Pt 3: 1066–1083. 10.1016/j.foodres.2016.08.016. 28865618

[crf370264-bib-0114] Ryu, J. , X. Xiang , X. Hu , et al. 2023. “Assembly of Plant‐Based Meat Analogs Using Soft Matter Physics: A Coacervation‐Shearing‐Gelation Approach.” Food Hydrocolloids 142: 108817. 10.1016/j.foodhyd.2023.108817.

[crf370264-bib-0115] Sadvakasova, A. K. , B. D. Kossalbayev , M. O. Bauenova , et al. 2023. “Microalgae as a Key Tool in Achieving Carbon Neutrality for Bioproduct Production.” Algal Research 72: 103096. 10.1016/j.algal.2023.103096.

[crf370264-bib-0116] Safi, C. 2013. “Microalgae Biorefinery: Proposition of a Fractionation Process.” Doctoral dissertation, INPT.

[crf370264-bib-0117] Safi, C. , M. Charton , A. V. Ursu , et al. 2014. “Release of Hydro‐Soluble Microalgal Proteins Using Mechanical and Chemical Treatments.” Algal Research 3: 55–60. 10.1016/j.algal.2013.11.017.

[crf370264-bib-0118] Safi, C. , G. Olivieri , R. P. Campos , et al. 2017. “Biorefinery of Microalgal Soluble Proteins by Sequential Processing and Membrane Filtration.” Bioresource Technology 225: 151–158. 10.1016/j.biortech.2016.11.068. 27888732

[crf370264-bib-0119] Safi, C. , A. V. Ursu , C. Laroche , et al. 2014. “Aqueous Extraction of Proteins From Microalgae: Effect of Different Cell Disruption Methods.” Algal Research 3: 61–65. 10.1016/j.algal.2013.12.004.

[crf370264-bib-0120] Sägesser, C. , J. M. Kallfelz , S. Boulos , et al. 2023. “A Novel Approach for the Protein Determination in Food‐Relevant Microalgae.” Bioresource Technology 390: 129849. 10.1016/j.biortech.2023.129849. 37813318

[crf370264-bib-0121] Sägesser, C. , J. M. Kallfelz , S. Boulos , et al. 2024. “Structurability of Microalgae, Soy and Pea Protein for Extruded High‐Moisture Meat Analogues.” Food Hydrocolloids, 156 156: 110290. 10.1016/j.foodhyd.2024.110290.

[crf370264-bib-0121a] Silva, F. A. , R. L. Dos Santos , C. E. Barão , et al. 2025. “Freshwater microalgae biomassesare a source of bioaccessible bioactive compounds and have antioxidant,antihypertensive, and antidiabetic activity.” Food Research International 208. 10.1016/j.foodres.2025.116259. 40263856

[crf370264-bib-0122] Water Science School . 2019. How Much Water is There on Earth? Water Science School.

[crf370264-bib-0123] Schwenzfeier, A. , A. Helbig , P. A. Wierenga , and H. Gruppen . 2013. “Emulsion Properties of Algae Soluble Protein Isolate From *Tetraselmis* sp.” Food Hydrocolloids 30, no. 1: 258–263. 10.1016/j.foodhyd.2012.06.002.

[crf370264-bib-0124] Schwenzfeier, A. , F. Lech , P. A. Wierenga , M. H. M. Eppink , and H. Gruppen . 2013. “Foam Properties of Algae Soluble Protein Isolate: Effect of pH and Ionic Strength.” Food Hydrocolloids 33, no. 1: 111–117. 10.1016/j.foodhyd.2013.03.002.

[crf370264-bib-0125] Schwenzfeier, A. , P. A. Wierenga , M. H. M. Eppink , and H. Gruppen . 2014. “Effect of Charged Polysaccharides on the Techno‐Functional Properties of Fractions Obtained From Algae Soluble Protein Isolate.” Food Hydrocolloids 35: 9–18. 10.1016/j.foodhyd.2013.07.019.

[crf370264-bib-0126] Schwenzfeier, A. , P. A. Wierenga , and H. Gruppen . 2011. “Isolation and Characterization of Soluble Protein From the Green Microalgae *Tetraselmis* sp.” Bioresource Technology 102, no. 19: 9121–9127. 10.1016/j.biortech.2011.07.046. 21831634

[crf370264-bib-0127] Shabaka, S. , and M. Moawad . 2021. “Ecology and Biochemical Composition of a Newly Reported Non‐Indigenous Red Alga, Grateloupia Gibbesii, in the Mediterranean Sea, With Reference to Edible Red Seaweeds.” Regional Studies in Marine Science 44: 101767. 10.1016/j.rsma.2021.101767.

[crf370264-bib-0128] Sheih, I.‐C. , T. J. Fang , and T.‐K. Wu . 2009. “Isolation and Characterisation of a Novel Angiotensin I‐Converting Enzyme (ACE) Inhibitory Peptide From the Algae Protein Waste.” Food Chemistry 115, no. 1: 279–284.

[crf370264-bib-0129] Shkolnikov Lozober, H. , Z. Okun , and A. Shpigelman . 2021. “The Impact of High‐Pressure Homogenization on Thermal Gelation of *Arthrospira platensis* (Spirulina) Protein Concentrate.” Innovative Food Science & Emerging Technologies 74: 102857. 10.1016/j.ifset.2021.102857.

[crf370264-bib-0130] Sikkema, A. 2021. Algae Cultivation in the Desert is Feasible .

[crf370264-bib-0131] Su, M. , L. Bastiaens , J. Verspreet , and M. Hayes . 2023. “Applications of Microalgae in Foods, Pharma and Feeds and Their Use as Fertilizers and Biostimulants: Legislation and Regulatory Aspects for Consideration.” Foods 12, no. 20: 3878. 10.3390/foods12203878. 37893770 PMC10606004

[crf370264-bib-0132] Suresh Kumar, K. , K. Ganesan , K. Selvaraj , and P. V. Subba Rao . 2014. “Studies on the Functional Properties of Protein Concentrate of *Kappaphycus alvarezii* (Doty) Doty—an Edible Seaweed.” Food Chemistry 153: 353–360. 10.1016/j.foodchem.2013.12.058. 24491740

[crf370264-bib-0133] Tangyu, M. , J. Muller , C. J. Bolten , and C. Wittmann . 2019. “Fermentation of Plant‐Based Milk Alternatives for Improved Flavour and Nutritional Value.” Applied Microbiology and Biotechnology 103, no. 23–24: 9263–9275. 10.1007/s00253-019-10175-9. 31686143 PMC6867983

[crf370264-bib-0134] Teuling, E. , J. W. Schrama , H. Gruppen , and P. A. Wierenga . 2019. “Characterizing Emulsion Properties of Microalgal and Cyanobacterial Protein Isolates.” Algal Research 39: 101471. 10.1016/j.algal.2019.101471.

[crf370264-bib-0135] Teuling, E. , P. A. Wierenga , J. W. Schrama , and H. Gruppen . 2017. “Comparison of Protein Extracts From Various Unicellular Green Sources.” Journal of Agricultural and Food Chemistry 65, no. 36: 7989–8002. 10.1021/acs.jafc.7b01788. 28701042 PMC5599872

[crf370264-bib-0136] UNICEF . 2023. The State of Food Security and Nutrition in the World 2023. UNICEF.

[crf370264-bib-0137] Urlass, S. , Y. Wu , T. T. L. Nguyen , P. Winberg , M. S. Turner , and H. Smyth . 2023. “Unravelling the Aroma and Flavour of Algae for Future Food Applications.” Trends in Food Science & Technology 138: 370–381. 10.1016/j.tifs.2023.06.018.

[crf370264-bib-0138] Ursu, A. V. , A. Marcati , T. Sayd , V. Sante‐Lhoutellier , G. Djelveh , and P. Michaud . 2014. “Extraction, Fractionation and Functional Properties of Proteins From the Microalgae *Chlorella vulgaris* .” Bioresource Technology 157: 134–139. 10.1016/j.biortech.2014.01.071. 24534795

[crf370264-bib-0140] Vásquez, V. , R. Martínez , and C. Bernal . 2019. “Enzyme‐Assisted Extraction of Proteins From the Seaweeds *Macrocystis pyrifera* and *Chondracanthus chamissoi*: Characterization of the Extracts and Their Bioactive Potential.” Journal of Applied Phycology 31, no. 3: 1999–2010. 10.1007/s10811-018-1712-y.

[crf370264-bib-0141] Vilatte, A. , X. Spencer‐Milnes , H. O. Jackson , S. Purton , and B. Parker . 2023. “Spray Drying is a Viable Technology for the Preservation of Recombinant Proteins in Microalgae.” Microorganisms 11, no. 2: 512. 10.3390/microorganisms11020512. 36838478 PMC9967251

[crf370264-bib-0142] Villaró, S. , G. Acién , J. M. Fernández‐Sevilla , and T. Lafarga . 2023. “Microalgal Protein Production: Current Needs and Challenges.” In Future Proteins, 153–171. Elsevier.

[crf370264-bib-0143] Waghmare, A. G. , M. K. Salve , J. G. LeBlanc , and S. S. Arya . 2016. “Concentration and Characterization of Microalgae Proteins From *Chlorella pyrenoidosa* .” Bioresources and Bioprocessing 3, no. 1: 16. 10.1186/s40643-016-0094-8.

[crf370264-bib-0144] Wang, M. , Z. Yin , W. Sun , Q. Zhong , Y. Zhang , and M. Zeng . 2023. “Microalgae Play a Structuring Role in Food: Effect of *Spirulina platensis* on the Rheological, Gelling Characteristics, and Mechanical Properties of Soy Protein Isolate Hydrogel.” Food Hydrocolloids 136: 108244. 10.1016/j.foodhyd.2022.108244.

[crf370264-bib-0145] Wang, M. , Z. Yin , and M. Zeng . 2022. “Microalgae as a Promising Structure Ingredient in Food: Obtained by Simple Thermal and High‐Speed Shearing Homogenization.” Food Hydrocolloids 131: 107743. 10.1016/j.foodhyd.2022.107743.

[crf370264-bib-0146] Wang, Y. , J. Zhao , W. Zhang , C. Liu , P. Jauregi , and M. Huang . 2020. “Modification of Heat‐Induced Whey Protein Gels by Basic Amino Acids.” Food Hydrocolloids 100: 105397. 10.1016/j.foodhyd.2019.105397.

[crf370264-bib-0147] Wells, M. L. , P. Potin , J. S. Craigie , et al. 2017. “Algae as Nutritional and Functional Food Sources: Revisiting Our Understanding.” Journal of Applied Phycology 29, no. 2: 949–982. 10.1007/s10811-016-0974-5. 28458464 PMC5387034

[crf370264-bib-0147a] WHO/FAO/UNU Expert Consultation . 2007. “Protein and amino acid requirements in human nutrition.” World Health Organization Technical Report 935: 1–265. 18330140

[crf370264-bib-0148] Wojciechowski, K. 2022. “Surface Tension of Native and Modified Plant Seed Proteins.” Advances in Colloid and Interface Science 302: 102641. 10.1016/j.cis.2022.102641. 35299137

[crf370264-bib-0149] Yang, S. , S. Fu , B. Liu , and K.‐W. Cheng . 2024. “High‐Pressure Homogenization Combined With Alcohol Treatment Is Effective in Improving the Sensory and Techno‐Functional Characteristics of Chlorella Pyrenoidosa.” Lwt 191: 115709. 10.1016/j.lwt.2023.115709.

[crf370264-bib-0150] Yap, B. H. J. , G. J. Dumsday , P. J. Scales , and G. J. O. Martin . 2015. “Energy Evaluation of Algal Cell Disruption by High Pressure Homogenisation.” Bioresource Technology 184: 280–285. 10.1016/j.biortech.2014.11.049. 25435068

[crf370264-bib-0151] Yucetepe, A. , O. Saroglu , C. Daskaya‐Dikmen , F. Bildik , and B. Ozcelik . 2018. “Optimisation of Ultrasound‐Assisted Extraction of Protein From *Spirulina platensis* Using RSM.” Czech Journal of Food Sciences 36, no. 1: 98–108. 10.17221/64/2017-cjfs.

[crf370264-bib-0152] Zhou, H. , G. Vu , and D. J. McClements . 2022. “Formulation and Characterization of Plant‐Based Egg White Analogs Using RuBisCO Protein.” Food Chemistry 397: 133808. 10.1016/j.foodchem.2022.133808. 35914453

[crf370264-bib-0153] Zhou, H. , B. Zheng , Z. Zhang , R. Zhang , L. He , and D. J. McClements . 2021. “Fortification of Plant‐Based Milk With Calcium May Reduce Vitamin D Bioaccessibility: An In Vitro Digestion Study.” Journal of Agricultural and Food Chemistry 69, no. 14: 4223–4233. 10.1021/acs.jafc.1c01525. 33787251

[crf370264-bib-0154] Žugčić, T. , R. Abdelkebir , F. J. Barba , et al. 2018. “Effects of Pulses and Microalgal Proteins on Quality Traits of Beef Patties.” Journal of Food Science and Technology 55, no. 11: 4544–4553. 10.1007/s13197-018-3390-9. 30333651 PMC6170368

